# Oligo-Fucoidan prevents IL-6 and CCL2 production and cooperates with p53 to suppress ATM signaling and tumor progression

**DOI:** 10.1038/s41598-017-12111-1

**Published:** 2017-09-19

**Authors:** Li-Mei Chen, Po-Yen Liu, Yen-An Chen, Hong-Yu Tseng, Pei-Chun Shen, Pai-An Hwang, Hsin-Ling Hsu

**Affiliations:** 1 Institute of Molecular and Genomic Medicine, National Health Research Institutes, Miaoli, Taiwan; 2 Department of Bioscience and Biotechnology, National Taiwan Ocean University, Keelung, Taiwan

## Abstract

Low-molecular-weight Fucoidan (Oligo-Fucoidan) is a sulfated polysaccharide that has a variety of biological effects and has also been shown to have beneficial health effects. However, the molecular mechanisms underlying the therapeutic effects of Oligo-Fucoidan in patients with cancer remain unclear. Using human colorectal cancer HCT116 cells with (p53^+/+^) or without (p53^−/−^) normal p53 expression, we found that Oligo-Fucoidan treatment reduces the occurrence of spontaneous DNA lesions. Etoposide induces double strand DNA breaks. Subsequent administration of Oligo-Fucoidan to etoposide-treated cells promotes p53 accumulation, p21 expression and significant decreases in ataxia-telangiectasia-mutated (ATM), checkpoint kinase 1 (Chk1) and γ-H2AX phosphorylation in p53^+/+^ cells compared with p53^−/−^ cells. Similarly, co-administration of Oligo-Fucoidan with etoposide inhibits ATM, Chk1 and γ-H2AX phosphorylation, particularly in the presence of p53. Furthermore, Oligo-Fucoidan supplementation increases cancer cell death and attenuates the adverse effects induced by etoposide that decreases production of the pro-inflammatory cytokine IL-6 and chemokine CCL2/MCP-1. Importantly, Oligo-Fucoidan decreases the tumor-promoting M2 macrophages in microenvironment as well as collaborates with p53 and works in combination with etoposide to prevent HCT116 tumorigenicity. Our results first demonstrate that p53 enables Oligo-Fucoidan to effectively inhibit tumor progression, and Oligo-Fucoidan minimizes the side effects of chemotherapy and alters tumor microenvironment.

## Introduction

Seaweeds contain many bioactive components of polysaccharides and polyphenols^1^, which exhibit therapeutic potential such as anti-cancer, anti-oxidant, anti-inflammatory and anti-diabetic effects^2^. Fucoidan is a sulfated fucose-rich polysaccharide isolated from brown seaweed and is under consideration for use as a functional food that can prevent disease and improve health^3–5^. In addition to exerting anti-oxidant^6^ and anti-inflammatory effects^7^, Fucoidan also increases cancer cell apoptosis^8,9^ and induces cell cycle arrest^10,11^.

Angiogenesis promotes tumor progression and metastasis^12^. Fucoidan treatment suppresses angiogenic activity by inhibiting vascular endothelial growth factor (VEGF) receptor expression and VEGF-induced human umbilical vein endothelial cell proliferation^13,14^. Matrix metalloproteinases (MMPs) and nuclear factor-kappa B (NF-κB), which regulate VEGF expression and hypoxia-inducible factor 1-alpha/VEGF signaling under hypoxic conditions, are also inhibited by Fucoidan treatment, which may prevent metastasis in tumor-bearing mice^15,16^.

Tumor microenvironment (TME) mediates tumor aggressiveness^17^. Fucoidan has anti-inflammatory effects that suppress nitric oxide synthase (iNOS), cyclooxygenase (COX)-2 and monocyte chemoattractant protein-1 (MCP-1/CCL2) expression, as well as pro-inflammatory cytokine production, namely, interleukin-1β (IL-1β) and tumor necrosis factor (TNF)-α production^7,18^. Moreover, Fucoidan has been shown to have immune-modulatory effects *in vivo* by enhancing natural killer (NK) cell and cytotoxic T-cell (CTL) numbers and activity^19–21^. A previous study showed that CTLs display greater cytotoxicity against cancer cells when co-cultured with Fucoidan-treated dendritic cells (DCs) than when cultured alone^22^. However, another study showed that Fucoidan induces expression of the anti-apoptotic protein Mcl-1, inactivates caspase-3 and activates the phosphoinositide 3-kinase/AKT signaling pathway to inhibit neutrophil apoptosis^23^.

As an antioxidant, Fucoidan protects cells against oxidative stress by scavenging superoxide radicals^24^; inducing expression of the anti-oxidant nuclear factor erythroid-2-related factor 2 and that of its target gene, superoxide dismutase^25^; and suppressing the transforming growth factor β (TGF-β)/Smad pathway^26^, which prevents reactive oxidative species (ROS) generation in cancer cells and ROS release into the TME^27^. These results suggest that Fucoidan modifies the TME.

The length of sugar backbone chain and the amount of sulfate groups affect the biological activity of Fucoidans. We have found that the low-molecular Fucoidan (LMF), high-molecular weight Fucoidan (HMF) and other Fucoidan derivatives from *Sargassum hemiphyllum* have different protective effects against ultraviolet B (UVB) damage on skin cells^28^. As compared with HMF, LMF more protects the skin cells against UVB damage and decreases collagen degradation by preventing the activation of transcription factor activator protein-1 (AP-1) which induces collagenases (MMP-1, MMP-8 and MMP-13) and gelatinase (MMP-9) but suppresses TGFβRII expression under UVB damage. Previous studies also indicated that LMF promotes TGF-β receptor degradation and induces Toll-like receptor 4-regulated reactive oxygen species that promotes endoplasmic reticulum stress-mediated apoptosis in lung cancer cells, thus inhibiting lung cancer progression^29,30^.

It is unclear to date whether low-molecular-weight Fucoidan (LMF) (also known as Oligo-Fucoidan) cooperates with tumor suppressor to further prevent tumor progression and change tumor microenvironment. Here, we first demonstrated that Oligo-Fucoidan collaborates with p53 to prevent spontaneous or etoposide-induced DNA breaks and to regulate the DNA damage response and cell cycle checkpoint. In addition to effectively reducing the side effects and enhancing the therapeutic results of etoposide, Oligo-Fucoidan also prevented tumor development in mice bearing HCT116 human colon cancer cells and suppressed M2 macrophage polarization. These results indicate that Oligo-Fucoidan is a promising supplement with respect to the treatment of cancer, as the compound enhances tumor suppressor activity, modulates cytokine profiles and alters tumor microenvironment.

## Results

### Oligo-Fucoidan and p53 prevent spontaneous DNA breaks in HCT116 cancer cells

**Figure 1:**
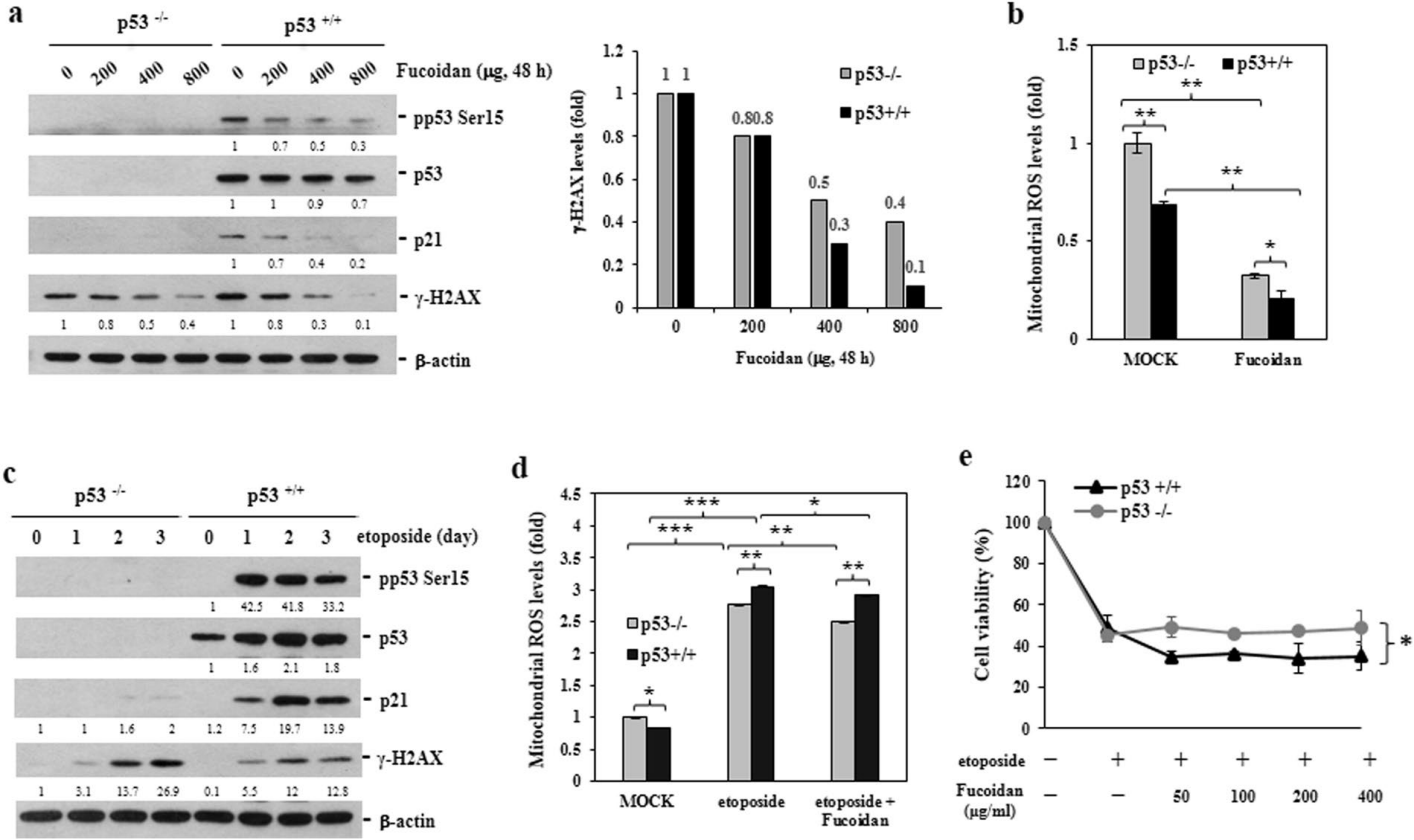
Oligo-Fucoidan prevents intrinsic DNA lesions and mitochondrial ROS generation. (**a**) HCT116 cell lines (p53^+/+^ and p53^−/−^) were treated with different doses of Oligo-Fucoidan for 48 h. p53, p21 and γ-HAX expression levels were analyzed in the indicated cells. γ-HAX levels were compared between p53^+/+^ and p53^−/−^ cells after Oligo-Fucoidan treatment. (**b**) Mitochondrial superoxide levels were detected by MitoSOX Red, followed by flow cytometry analysis, in cells treated with PBS (MOCK) or Oligo-Fucoidan (400 μg/ml) for 48 h. (**c**) p53, p21 and γ-HAX expression levels were studied in cells exposed to etoposide (40 μM) for different intervals. The protein levels were normalized to those β-actin, and the levels of their corresponding controls were set as 1. (**d**) Mitochondrial superoxide levels were measured after etoposide (40 μM) and Oligo-Fucoidan (400 μg/ml) administration or etoposide treatment alone for 48 h. (**e**) Cell viability was analyzed after the indicated cells being treated with etoposide alone or co-treated with different concentrations of Oligo-Fucoidan for 48 h. The data represent the mean ± SD of three independent experiments. *p < 0.05; **p < 0.01; ***p < 0.001.

Two isogenic HCT116 colorectal cancer cell lines without (p53^−/−^) or with (p53^+/+^) normal p53 expression were used to compare the effects of monotherapy with those of simultaneous or sequential combination therapy. When the p53^−/−^ and the p53^+/+^ cells were treated with different concentrations of Oligo-Fucoidan for 48 h (Fig. 1a), the phosphorylation (Ser139) of Histone H2AX (γ-H2AX), a biomarker for DNA double-strand breaks (DSBs), decreased significantly in a dose-dependent manner particularly in the p53^+/+^ cells. Oligo-Fucoidan also suppressed p53 (Ser15) phosphorylation and p21 expression. These effects imply that Oligo-Fucoidan protects the cells against intrinsic DNA lesions.

Mitochondrial ROS induce DNA breaks and gene mutations that participate in oncogenic pathways^31^. We detected mitochondrial ROS production by oxidative MitoSOX red and flow cytometry analysis and found that p53^+/+^ cells produced less mitochondrial superoxide than p53^−/−^ cells and that ROS production was repressed further after Oligo-Fucoidan (400 μg/ml) treatment for 48 h (Fig. 1b), suggesting that Oligo-Fucoidan and p53 may collaborate to attenuate oxidative DNA damage.

As a result of treatment with the chemoagent etoposide (VP-16) (40 μM) for different intervals (Fig. 1c), p53 accumulation increased significantly and accompanied by p21 induction in p53^+/+^ cells. However, γ-H2AX phosphorylation was increased to a lesser extent in p53^+/+^ cells than p53^−/−^ cells, indicating that p53 protected the cells from the genotoxicity of etoposide.

Excessive mitochondrial ROS generation disrupts mitochondrial function^32^, leading to ATP exhaustion and cell death. Etoposide induces cell death via p53-mediated processes in mitochondria^33^. We found that etoposide treatment for 48 h dramatically elevated mitochondrial superoxide levels in p53^−/−^ cells (2.75-fold, p < 0.001) and p53^+/+^ cells (3.04-fold, p < 0.001) compared with MOCK treatment groups (Fig. 1d), but the elevated mitochondrial ROS levels were moderately reduced by Oligo-Fucoidan supplementation. Cell viabilities were also reduced approximately by 54.8% in p53^−/−^ cells and by 51.2% in p53^+/+^ cells treated with etoposide for 48 h and further reduced by 65.4% in p53^+/+^ cells co-treated with Oligo-Fucoidan and etoposide (Fig. 1e), as determined by MTT analysis. Consequently, the reductions in mitochondrial ROS generation elicited by adjuvant Oligo-Fucoidan treatment are not sufficient to inhibit etoposide-induced cytotoxicity.

### Oligo-Fucoidan and p53 cooperatively regulate the DNA damage response

**Figure 2:**
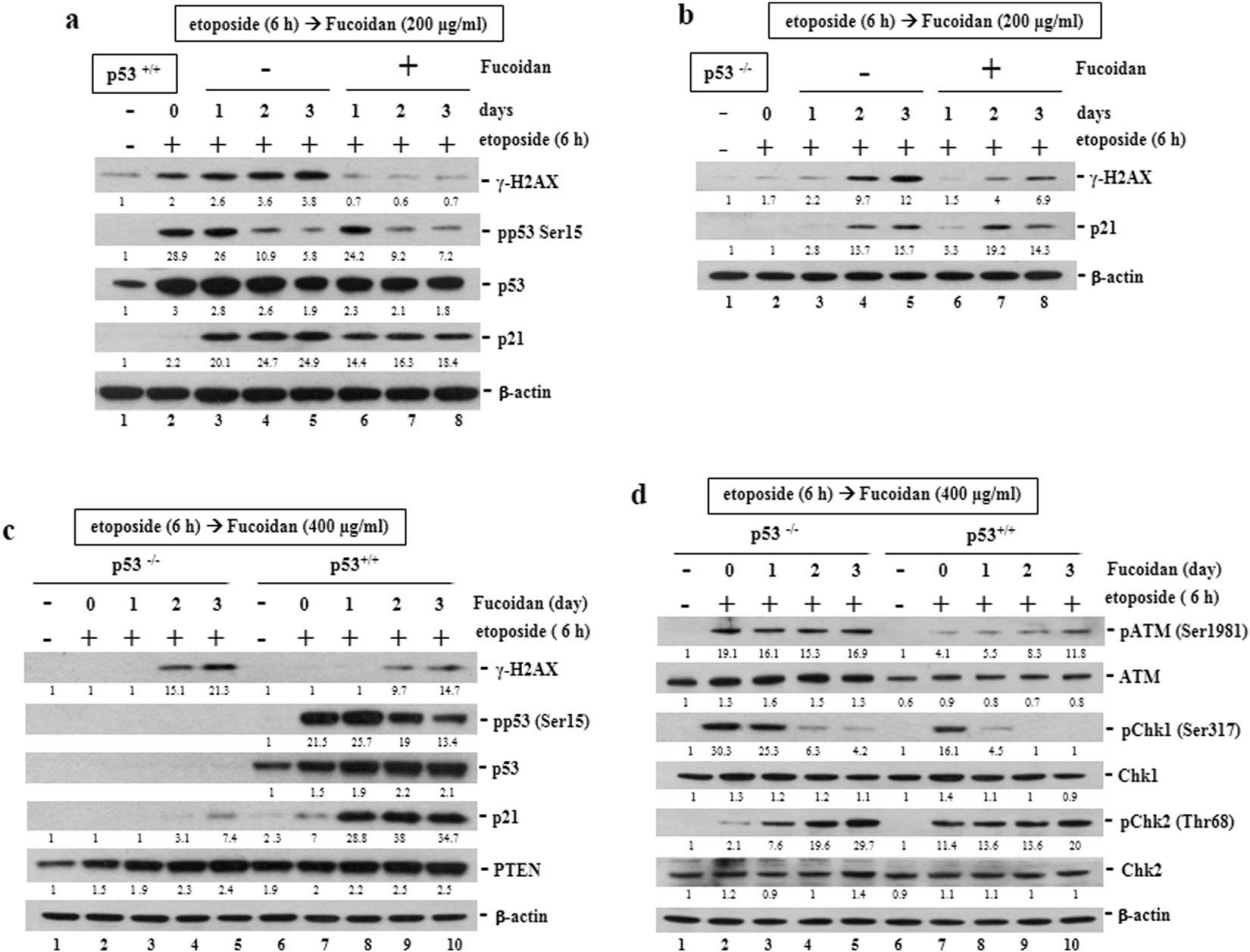
Oligo-Fucoidan and p53 cooperate to protect HCT116 cells against genotoxicity. p53, p21 and γ-H2AX expression levels were analyzed after p53^+/+^ (**a**) and p53^−/−^ (**b**) HCT116 cells were exposed to etoposide (40 μM) for 6 h before being treated with Oligo-Fucoidan (200 μg/ml) for different intervals (0–3 days). HCT116 cells were exposed to etoposide (40 μM) for 6 h before being treated with a higher dose of Oligo-Fucoidan (400 μg/ml) for different periods (0–3 days) (c-d). γ-H2AX, p53, p21 and PTEN expression levels were subsequently analyzed (**c**). ATM, Chk1 and Chk2 signaling activation was assessed (**d**). The protein levels were normalized to those β-actin, and the levels of their corresponding controls were set as 1.

We assessed the cells treated sequentially with etoposide (40 μM) for 6 h and then Oligo-Fucoidan (200 μg/ml) for different intervals (0–3 days). We found that Oligo-Fucoidan supplementation significantly reduced γ-H2AX levels in p53^+/+^ cells after etoposide treatment (Fig. 2a, lanes 6–8) compared with p53^+/+^ cells treated with etoposide alone (lanes 2–5). We also found that Oligo-Fucoidan attenuated etoposide-induced increases in γ-H2AX levels in p53^−/−^ cells (Fig. 2b, lanes 6–8) compared with MOCK-treated group (lanes 3–5). These findings show that Oligo-Fucoidan supplementation protects cells against etoposide-related genotoxicity, particularly in the presence of p53.

When HCT116 cells were exposed to a higher dose of Oligo-Fucoidan (400 μg/ml) for different intervals after 6 h of etoposide treatment (Fig. 2c), p53 accumulation and phosphorylation levels significantly increased in p53^+/+^ cells, changes accompanied by p21 induction (lanes 8–10). Again, γ-H2AX levels were lower in p53^+/+^ cells than p53^−/−^ cells treated with Oligo-Fucoidan (lanes 9–10 vs. 4–5). Differently, the tumor suppressor PTEN levels were constitutively induced in p53^+/+^ cells and p53^−/−^ cells treated sequentially with etoposide and Oligo-Fucoidan.

When assessed ATM signaling pathway activity in different p53 backgrounds (Fig. 2d), we found that Oligo-Fucoidan (400 μg/ml) supplementation significantly inhibited ATM (Ser1981) and Chk1 (Ser317) phosphorylation and slightly reduced Chk2 (Thr68) phosphorylation in p53^+/+^ cells (lanes 8–10) compared with p53^−/−^ cells sequentially treated with both agents (lanes 3–5). Similar results were obtained in the experiments in which the cells were treated with Oligo-Fucoidan (200 μg/ml) and etoposide simultaneously for different intervals (0–3 days) (Supplementary Fig. 1), as ATM, Chk1 and H2AX phosphorylation levels were all significantly reduced in p53^+/+^ cells compared with p53^−/−^ cells treated simultaneously. Taken together, these findings show that the ATM signaling cascade is jointly inhibited by Oligo-Fucoidan and p53.

Furthermore, the efficacy of high molecular weight Fucoidan (20–200 kDa) purified from *Fucus vesiculosus* was studied and compared with Oligo-Fucoidan (LMF) (0.5–0.8 kDa) from *Sargassum hemiphyllum* in regulation of DNA damage response (Supplementary Fig. 2). In response to different doses (0–800 μg/ml) of the HMF (*Fucus vesiculosus*) treatment for 48 h, spontaneous DNA breaks in the p53^+/+^ HCT116 cells were only reduced by the HMF at a higher concentration (800 μg/ml) (Supplementary Fig. 2a). As compared with LMF, the HMF (400 μg/ml) showed smaller effects on inhibiting γ-H2AX level and ATM phosphorylation but still promoted PARP cleavage in the p53^−/−^ cells co-treated with etoposide for 48 h (Supplementary Fig. 2b). Similarly, the γ-H2AX level and ATM phosphorylation in the p53^+/+^ cells were more decreased by treatment with LMF than HMF (Supplementary Fig. 2c). Thus, Oligo-Fucoidan (LMF) and the HMF have differential results on preventing spontaneous DNA breaks and on regulating DNA damage response.

**Figure 3:**
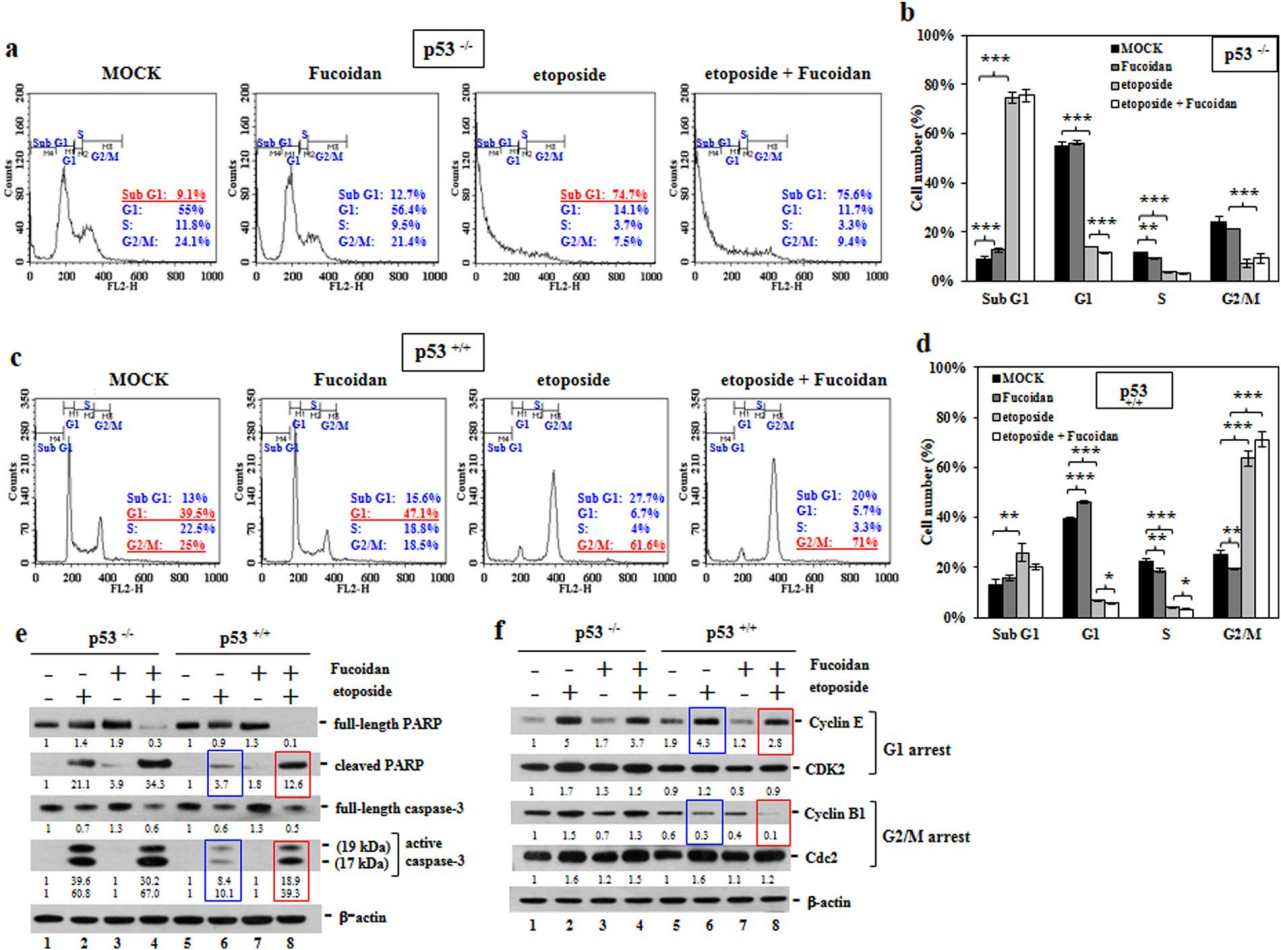
Oligo-Fucoidan and p53 cooperate to regulate DNA damage checkpoints. HCT116 cells were treated with PBS, Oligo-Fucoidan (400 μg/ml) and/or etoposide (40 μM) for 48 h. (**a**) The p53^−/−^ cell cycle profile was characterized. (**b**) Histograms reveal comparisons of the p53^−/−^ cell cycle profiles under different experimental settings. (**c**) The p53^+/+^ cell cycle profile was assessed. (**d**) Histograms display comparisons of the p53^+/+^ cell cycle profiles under different treatment conditions. (**e**) The levels of the indicated apoptotic markers (cleaved PARP and active caspase 3) and their intact molecules were examined. (**f**) The molecules responsible for regulating the G1 and G2/M checkpoints were characterized. The data represent the mean ± SD of three independent experiments. *p < 0.05; **p < 0.01; ***p < 0.001. The protein levels were normalized to those β-actin, and the levels of their corresponding controls were set as 1.

We subsequently evaluated the changes in the cell cycle profile induced by Oligo-Fucoidan (400 μg/ml) and/or etoposide treatment for 48 h. We found that Oligo-Fucoidan alone did not significantly alter the cell cycle profiles in p53^−/−^ cells (Fig. 3a); however, etoposide induced a significant increase in the size of the sub-G1 population (74.7%) in the corresponding group compared with MOCK treatment group (9.1%), a change indicative of increased apoptosis. Histograms indicating the cell cycle profiles altered by the indicated treatments are shown in the specified study (Fig. 3b).

In the p53^+/+^ cell experiments (Fig. 3c), Oligo-Fucoidan-treated cells experienced more G1 arrest (47.1%), and etoposide-treated cells experienced significantly more G2/M arrest (61.6%) than MOCK treatment. Oligo-Fucoidan and etoposide-treated cells experienced greater G2/M arrest (71%) than their counterparts. Histograms reveal the cell cycle profiles changed by the indicated treatments shown in the designated results (Fig. 3d). These findings indicate that Oligo-Fucoidan collaborates with p53 to arrest cells at the G1 and G2/M phases.

We investigated whether Oligo-Fucoidan affects chemosensitivity and found that expression of the cleaved PARP (Asp175) and active caspase 3 (Fig. 3e, lanes 4 and 8), indicators of pro-apoptotic effects, were significantly induced by simultaneous etoposide and Oligo-Fucoidan treatment for 48 h, showing that Oligo-Fucoidan improved therapeutic outcomes in etoposide-treated groups compared with cells treated with etoposide alone (lanes 2 and 6). The p53-p21 signaling pathway controls the G1 and G2/M phase checkpoints by inhibiting Cyclin E-Cdk2 (cyclin-dependent kinase 2) and Cyclin B-Cdc2 activity^34^. The Cdc2/Cyclin B1 pathway is inhibited by doxorubicin, which induces G2/M arrest in p53 wild-type cancer cells^35^. Consistent with these results, our data revealed that Oligo-Fucoidan supplementation decreased Cyclin E (G1 marker) and Cyclin B1 (mitotic inducer) levels, particularly in p53^+/+^ cells exposed to etoposide (Fig. 3f, lane 4 vs. 8), highlighting that Oligo-Fucoidan and p53 collaborate to enhance G1 and G2/M checkpoint activity.

### Oligo-Fucoidan inhibits CCL2 and IL-6 secretion, as well as STAT3 and STAT5 activation

**Figure 4:**
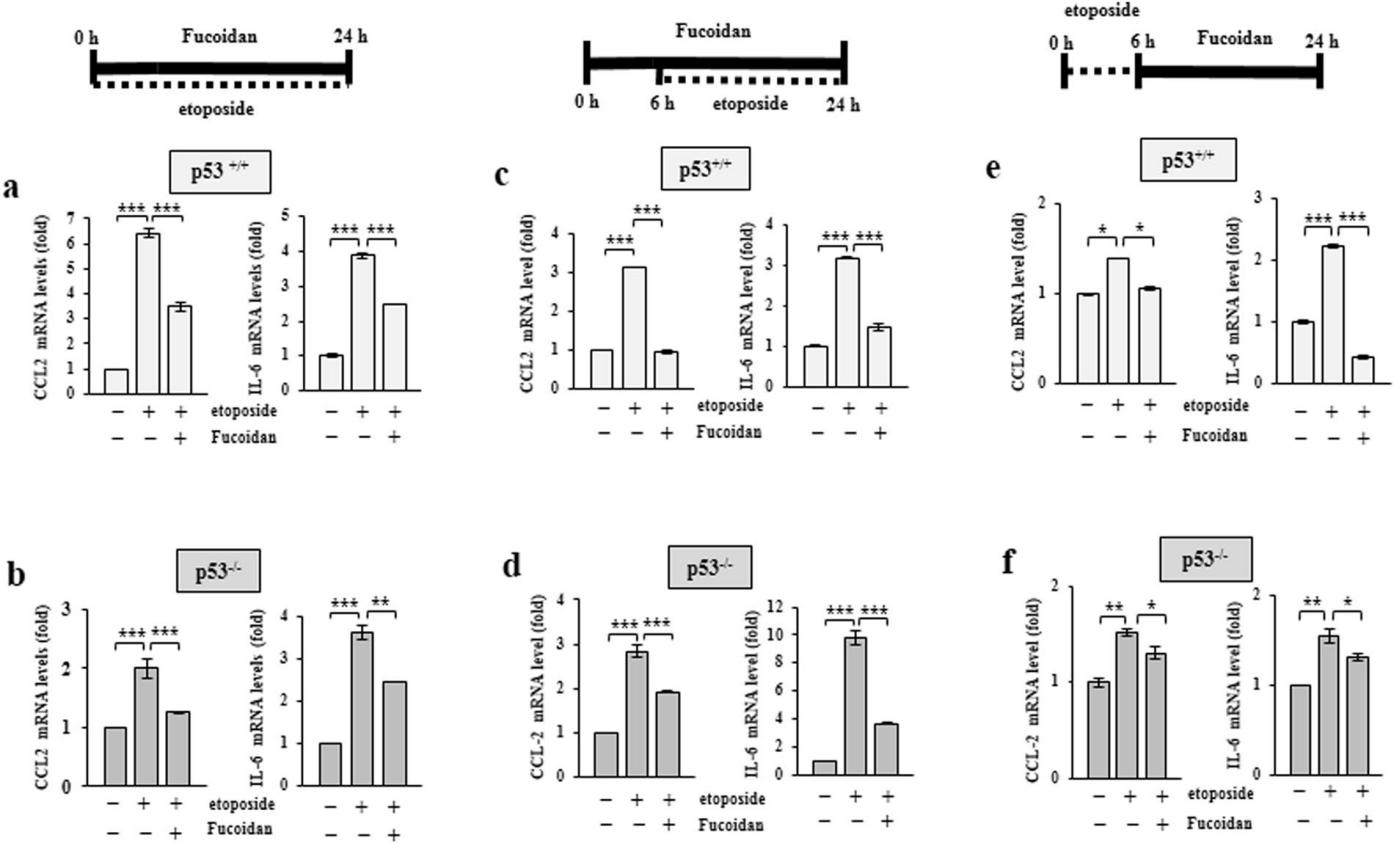
Oligo-Fucoidan reduces CCL2 and IL-6 expression. CCL2 and IL-6 mRNA expression levels were examined in the p53^+/+^ (**a**,**c**,**e**) and p53^−/−^ (**b**,**d**,**f**) cells. Cells treated with etoposide (40 μM) alone or etoposide and Oligo-Fucoidan (400 μg/ml) for 24 h were studied (**a**,**b**). The cells were pre-incubated with or without Oligo-Fucoidan for 6 h before being treated with etoposide for another 18 h (**c**,**d**). The cells were analyzed after being exposed to etoposide for only 6 h before being treated with Oligo-Fucoidan for another 18 h (**e**,**f**). The data represent the mean ± SD of three independent experiments. *p < 0.05; **p < 0.01; ***p < 0.001.

To investigate whether Oligo-Fucoidan affects chemotherapy side effect profiles, HCT116 cells were treated with etoposide and/or Oligo-Fucoidan (400 μg/ml) for 24 h (Fig. 4a,b). We found that etoposide increased the mRNA expression levels of the pro-inflammatory chemokine CCL2 by 6.4-fold in p53^+/+^ cells compared with MOCK-treated cells (Fig. 4a); however, we also found that Oligo-Fucoidan decreased CCL2 mRNA expression levels by 3.3-fold in etoposide-treated p53^+/+^ cells. Similarly, etoposide increased the mRNA expression levels of the pro-inflammatory cytokine IL-6 by 3.9-fold in p53^+/+^ cells compared with MOCK-treated cells; however, Oligo-Fucoidan decreased IL-6 mRNA expression levels by 2.5-fold in etoposide-treated p53^+/+^ cells. The inhibition effects of Oligo-Fucoidan on the above mentioned etoposide-induced increases in CCL2 and IL-6 mRNA expression levels were also noted in p53^−/−^ cells (Fig. 4b).

Furthermore, when p53^+/+^ cells were pre-treated with Oligo-Fucoidan (400 μg/ml) for 6 h before being treated with etoposide for another 18 h (Fig. 4c), both CCL2 and IL-6 mRNA levels increased by more than 3-fold in etoposide-treated cells compared with MOCK-treated cells; however, these increases were all significantly repressed by Oligo-Fucoidan and etoposide co-treatment. Similar results were also identified in p53^−/−^ cells treated simultaneously with Oligo-Fucoidan and etoposide (Fig. 4d). In cells treated with etoposide for only 6 h before being treated with Oligo-Fucoidan for 18 h (Fig. 4e,f), CCL2 and IL-6 mRNA expression was also repressed in a p53-independent manner. Therefore, Oligo-Fucoidan supplementation effectively inhibits CCL2 and IL-6 mRNA expression after simultaneous and sequential treatment of etoposide, indicating that Oligo-Fucoidan can reduce the adverse effects of etoposide.

**Figure 5:**
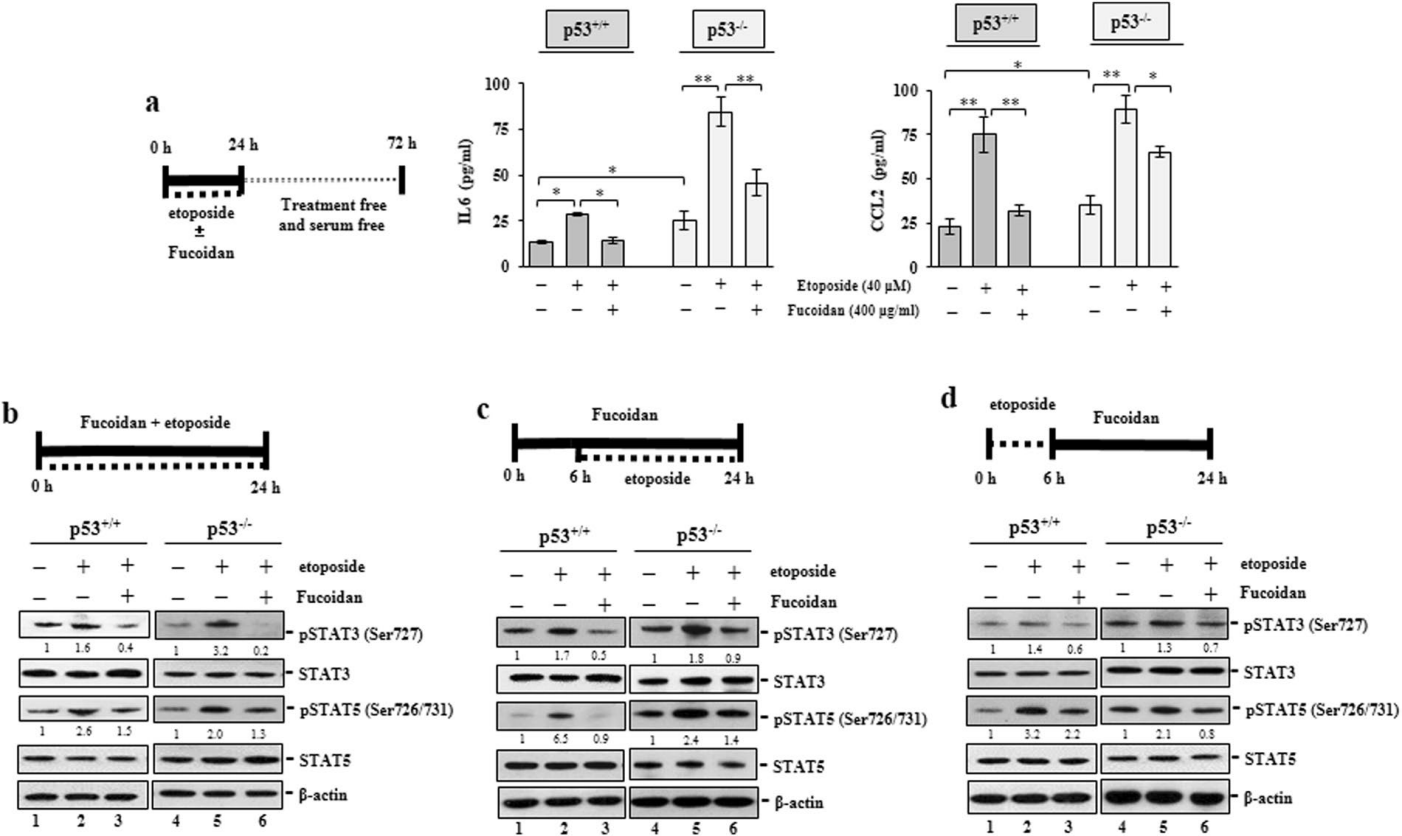
Oligo-Fucoidan suppresses IL-6 and CCL2 production as well as STAT3 and STAT5 activation. (**a**) HCT116 cells were treated with etoposide (40 μM) alone or etoposide and Oligo-Fucoidan (400 μg/ml) for 24 h. IL-6 and CCL2 secretion was measured after the cells were incubated in treatment-free and serum-free medium for 48 h. The results represent the mean ± SD of three independent experiments. *p < 0.05; **p < 0.001. (**b**) STAT3 (Ser727) and STAT5 (Ser726/731) phosphorylation was analyzed after the above cells were treated with etoposide alone or Oligo-Fucoidan and etoposide for 24 h. (**c**) The cells were studied after being treated with etoposide for 18 h or pre-treated with Oligo-Fucoidan for 6 h before being treated etoposide for another 18 h. (**d**) The cells were characterized after etoposide treatment for only 6 h or followed by Oligo-Fucoidan treatment for 18 h. STAT3 (Ser727) and STAT5 (Ser726/731) phosphorylation levels were individually normalized to total STAT3 and STAT5 levels. The corresponding expression levels of STAT3 and STAT5 in the MOCK-treated group were defined as 1.

To further examine the impact of Oligo-Fucoidan on IL-6 and CCL2 secretion from cancer cells, we treated HCT116 cells with etoposide and Oligo-Fucoidan (400 μg/ml) or etoposide alone for 24 h before incubating the cells in serum-free media for another 48 h (Fig. 5a). We found that Oligo-Fucoidan prevented etoposide-induced IL-6 and CCL2 release from the p53^+/+^ cells and p53^−/−^ cells, as analyzed IL-6 and CCL2 concentrations in condition medium by the quantitative ELISA. The etoposide-promoted IL-6 and CCL2 secretion from HCT116 cancer cells were also particularly prevented by Oligo-Fucoidan in the presence of p53.

CCL2 and IL-6 induce each other and cooperate to activate STAT3-Twist signaling and promote the polarization of monocytes into M2 macrophages^36^. STAT3 participates in a feedback loop, in which it stimulates the production of IL-6 and CCL2, thereby promoting Twist expression to enhance epithelial-mesenchymal transition (EMT). Furthermore, CCL2 promotes vascular permeability, extravasation and metastasis via JAK2-STAT5 and MAPK signaling^37^. To determine whether Oligo-Fucoidan affects STAT3 and STAT5 activation, HCT116 cells were treated synchronously with Oligo-Fucoidan (400 μg/ml) and etoposide or etoposide alone for 24 h (Fig. 5b), we found that STAT3 (Ser727) and STAT5 (Ser726/731) phosphorylation was enhanced by etoposide in the p53^+/+^cells and the p53^−/−^ cells (lanes 2 and 5) but was repressed by Oligo-Fucoidan supplementation (lanes 3 and 6). Moreover, in cells pre-treated with Oligo-Fucoidan for 6 h before being treated with etoposide for another 18 h (Fig. 5c), STAT3 and STAT5 phosphorylation levels (lanes 2 and 5) were again reduced by Oligo-Fucoidan co-treatment (lanes 3 and 6). Similarly, STAT3 and STAT5 phosphorylation was repressed in cells exposed to etoposide for only 6 h before being treated with Oligo-Fucoidan for 18 h, (Fig. 5d, lanes 3 and 6). Hence, Oligo-Fucoidan significantly prevents the inflammatory factors secreted from cancer cells and the corresponding signaling pathways in cancer cells, which may alter the development of the TME.

### Oligo-Fucoidan prevents M2 macrophage polarization and cooperates with p53 to attenuate tumor progression

**Figure 6:**
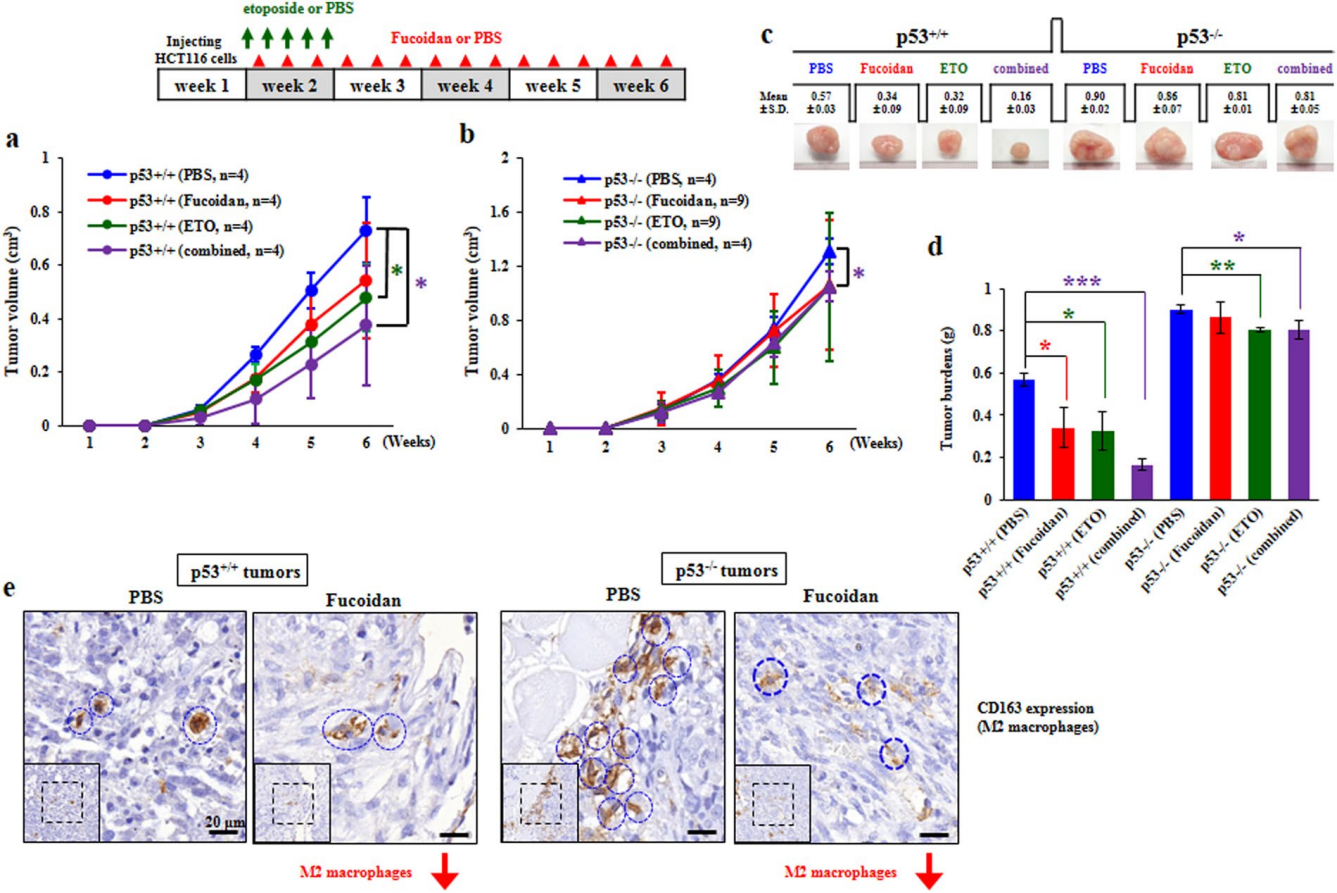
Oligo-Fucoidan cooperates with p53 and works with etoposide to further inhibit HCT116 tumor growth. After nude mice injecting HCT116 cells for 1 week, the mice were i.p. injected with etoposide (8 mg/kg) or PBS for 5 consecutive days. Afterward, the mice were fed with Oligo-Fucoidan (300 mg/kg) or PBS orally thrice a week for 5 weeks. The effects of single and combination therapies on tumor growth of the p53^+/+^ cell-bearing mice (**a**) and the p53^−/−^ cell-bearing mice (**b**) were assessed. The results of HCT116 tumor burdens after different treatments were analyzed (**c**). Histograms show the therapeutic effects of the indicated treatments in HCT116 tumor-bearing mice (**d**). The tumor-promoting M2 macrophages (indicated by dashed circles) identified in TME of the mice-bearing p53^+/+^ and p53^−/−^ tumors were examined by immunohistochemistry of CD163 expression (**e**). Scale bar represents 20 μm.

To investigate whether Oligo-Fucoidan proficiently prevents HCT116 tumor growth and enhances the therapeutic effect of etoposide, we subcutaneously injected BALB/c nude mice with HCT116 cells (2 × 10^6^). One week after HCT116 cell inoculation, the xenograft mice were i.p. injected with etoposide (8 mg/kg, 1/5 of the maximum-tolerated dose) or PBS for 5 consecutive days and fed Oligo-Fucoidan (150 mg/kg) or PBS thrice weekly for 5 weeks. Importantly, we found that tumor growth was attenuated in p53^+/+^ cell-bearing mice treated with Oligo-Fucoidan and etoposide simultaneously or etoposide alone (Fig. 6a). However, tumor growth rates were moderately decreased only in p53^−/−^ cell-bearing mice simultaneously treated with Oligo-Fucoidan and etoposide (Fig. 6b). After 6 weeks of cancer cell inoculation, we measured p53^+/+^ tumor loads and found that they were much smaller than p53^−/−^ tumor loads (Fig. 6c) and that p53^+/+^ tumor burdens were significantly decreased not only by treatment with Oligo-Fucoidan or etoposide alone but also further suppressed by combination treatment with both agents. However, co-treatment with Oligo-Fucoidan and etoposide decreased p53^−/−^ tumor burdens only moderately. Histograms showing the differential therapeutic effects are identified in the indicated treatments of HCT116 tumor (Fig. 6d). These are the first results demonstrating that Oligo-Fucoidan and etoposide co-treatment exhibits better therapeutic performance in the presence of p53 than in the absence of p53.

We conducted further surveillance of the TME by performing immunohistochemical staining analysis of the tumor-associated M2 macrophage marker CD163 (indicated by dashed circles) in the above tumors (Fig. 6e). We found that p53^+/+^ tumors exhibited less numbers of M2 macrophages accumulated in the microenvironment than p53^−/−^ tumors displayed highly enriched M2 macrophages, whereas Oligo-Fucoidan treatment significantly reduced M2 populations in p53^+/+^ tumor and particular in p53^−/−^ tumor, revealing that Oligo-Fucoidan can alter the TME development independent of p53 status.

## Discussion

Here, we demonstrated that Oligo-Fucoidan proficiently enhances DNA damage checkpoint activity, improves therapeutic results and reduces the adverse effects of etoposide on colorectal cancer cells. Oligo-Fucoidan collaborates with p53 to decrease the ATM signaling pathway activity and to significantly suppress tumor growth. Importantly, Oligo-Fucoidan inhibits the effects of etoposide on promoting IL-6 and CCL2 signaling function and decreases M2 macrophage recruitment in TME. The IL-6/CCL2/EMT axis stimulates tumor progression and metastasis^38^, suggesting that Oligo-Fucoidan supplementation may prevent tumor metastasis or even relapse.

Different from the effect of Oligo-Fucoidan on suppressing mitochondrial ROS generation in HCT116 cancer cells (Fig. 1b), Fucoidan enhances the endoplasmic reticulum stress cascade which triggers apoptosis of highly metastatic cancer cells and also decreases tumor progression and metastasis^11,39,40^. Oligo-Fucoidan also attenuates PDGF-induced cell proliferation and induces G_1_/G_0_ cell cycle arrest^31^. We demonstrated that p53 is a key facilitator of the ability of Oligo-Fucoidan to promote G1 and G2/M checkpoint activity (Fig. 3c) and exert therapeutic effects on HCT116 tumors in the response to etoposide (Fig. 6). The suppressive effects of Oligo-Fucoidan on Cyclin E (CDK2 activator) and Cyclin B1 (mitotic activator) enable to increase cell cycle arrest at the G1 and G2/M phases in the p53^+/+^ cells treated with etoposide (Fig. 3c,f).

The ATM-dependent DNA damage response contributes to the repair of DSBs in higher eukaryotic cells and cancer cells^41,42^. Mis-repair of radiotherapy or chemotherapy-induced DSBs leads to robust chromosomal aberrations or cancer cell cytotoxicity. However, DNA repair machinery is highly activated in aggressive tumor cells and thus confers resistance to radiotherapy and chemotherapy. Therefore, agents that damage DNA repair machinery can advance cancer cell death and prevent treatment-resistant stemness. Oligo-Fucoidan impedes etoposide inducing the rapid activation of ATM and its downstream molecules (Chk1 and Chk2) (Fig. 2d), which may enhance chemosensitivity and lead to the reduced chances of cancer stemness and relapse.

Low-molecular-weight Fucoidan (LMF) (also known as Oligo-Fucoidan and LMW Fucoidan) has been found to attenuate the adverse effects of gemcitabine and cisplatin and to inhibit cancer cachexia-related muscle atrophy during chemotherapy^43^, which induces NF-κB, myostatin and activin A expression. LMF not only minimized cachexia-related symptoms but also repressed lung carcinoma metastasis by inhibiting VEGF and MMPs in tumor-bearing mice^15^. In addition, LMF regulates the miR-29b-DNMT3B-MTSS1 axis in hepatocellular carcinoma cells^44^. LMF also attenuates tumor development and angiogenesis in mice^45^; suppresses the TGF-β receptor (TGFR)/Smad/Snail/Slug and EMT axes^39^; and inhibits the TGFR/Smad7/Smurf2 axis in lung cancer cells^29^.

Cytokine and chemokine overproduction promotes cancer cell proliferation, invasion and angiogenesis that confers resistance to therapy and facilitates tumor progression^41,46,47^. Cancer cells resistant to chemotherapy and oncoprotein-targeted drugs display increased IL-1α, IL-6, IL-8, TNF-α, HGF, EGF, VEGF, GM-CSF and SDF-1α expression^47^. Tumor irradiation also induces TGF-β and IL-6 expression, thereby triggering the ATM-dependent DNA damage response and repair pathway in cancer cells^41^. Moreover, patients with pancreatic cancer with high IL-6 and IL-1β levels display low overall and recurrence-free survival after gemcitabine treatment^48^. Therefore, Oligo-Fucoidan supplementation promotes cancer cell death (Figs 1e and 3e) and inhibits the ATM signaling activity (Fig. 2 and Supplementary Fig. 1), an effect that may be attributable to inhibition of IL-6 activity.

IL-6 expression induced by etoposide activates the JAK1-STAT3 pathway which mediates anti-senescence and promotes tumor growth^49^. Upregulated IL-6 inhibits p53, TGF-β and etoposide-induced apoptosis in cancer cells^50^. Simultaneous Oligo-Fucoidan and etoposide treatment reduces CCL2 and IL-6 secretion and signaling (Fig. 5), which also explain the enhanced effects of p53 accumulation and HCT116 cell apoptosis (Figs 1e and 2c). Healthy mice treated with etoposide experience rapid increases in their serum IL-6 levels and develop chemotherapy-related illnesses^51^, whose symptoms and associated fatigue resemble those of cancer. We speculate that Oligo-Fucoidan may be able to attenuate etoposide-induced side-effects, distress and inflammation in affected patients. The IL-6/IL-6R/STAT3 pathway mediates interactions between tumor cells and the TME and contributes to the development of drug resistance^52^. By modulating the immune and cytokine systems, Oligo-Fucoidan may prevent autocrine loops in cancer cells and paracrine pathways in microenvironmental cells, thereby improving therapeutic efficacy and overall survival rate.

Although etoposide treatment does not influence tumor-associated M1 or M2 macrophage polarization^53^, it significantly promotes apoptosis in HepG2 hepatoma cells or A549 lung adenoma cells co-cultured with M1 macrophages but not M2 macrophages derived from the THP-1 monocytes. Oligo-Fucoidan treatment is sufficient to decrease M2 macrophage differentiation in TME independent of p53 status (Fig. 6e). It will be important to investigate whether Oligo-Fucoidan impacts the classical activation of M1 macrophages and/or the inhibition of M2 macrophages via inhibiting CCL2 and IL-6 signaling, which modulate anti-inflammation, tumor immunity and tissue remodeling (angiogenesis and metastasis).

The inhibitory effects of LMF (Oligo-Fucoidan) on cancer cell progression have been well-characterized, but the direct target(s) or interacting protein(s) of LMF which determine LMF biological activity are unclear. Toll-like receptor 4 (TLR4), an membrane-bound receptor, may serve as a binding site and signaling transducer of LMF which rapidly induces ROS-mediated endoplasmic reticulum (ER) stress and then activates the PERK-ATF4-CHOP pathway to trigger lung cancer cell death and suppress tumor progression^30^. Moreover, LMF targets transforming growth factor β (TGFβ) receptors (TGFRs) and degrades TGFRs via the Smurf2-dependent ubiquitin-proteasome pathway, leading to the inhibition of Smad2/3 and non-Smad pathways: AKT, ERK1/2 and FAK phosphorylation which are implicated in lung cancer cell progression *in vitro* and *in vivo* ^29^. Our findings support that p53 is an important intracellular counterpart of Oligo-Fucoidan that benefits to prevent DNA breaks, the adverse effect of chemotherapy, colon cancer growth and M2 macrophage polarization *in vivo*. Clearly, LMF performs multiple tasks for execution of anti-cancer functions.

Intriguingly, LMF also helps treatment of renal tubulointerstitial fibrosis^54^, a key determinant of progressive chronic kidney disease (CKD). The results showed that less than 100 mg/kg/d of LMF treatment improved renal function and reduced renal tubulointerstitial fibrosis in CKD mice. Importantly, LMF inhibited pressure-induced fibrotic response and the expression of CD44, β-catenin and TGF-β in rat renal tubular cells. However, adding CD44 ligands counteracted the anti-fibrotic effect of LMF, suggesting that LMF may directly interfere with the interaction between CD44 and extracellular ligands to decrease renal tubulointerstitial fibrosis.

Previous studies have reported that LMF and the high molecular weight Fucoidan from *Fucus vesiculosus* show similar results in anti-lung cancer^30^ and anti-DNA lesions at higher concentrations (Supplementary Fig. 2a), even though they are isolated from different species of brown seaweeds. We propose that LMF and the HMF may have similar structural characteristics. Although the exact structure of LMF is under investigation, based on the molecular weights and sulfate contents of LMF, we speculate that LMF probably contains 5~6 sugar residues and that are sulfated mostly at C-2 and/or C-4 comparable to the structure of Fucoidans as described previously^3,55–58^. LMF may mainly contain an α-1,3-linked fucose backbone or a repeat disaccharide unit containing α-1,3-linked fucose and α-1,4-linked fucose with sulfate attached at the C-2 and/or C-4 positions. The relationship of different structural features of Fucoidans and biological activities are certainly worth further investigation.

## Methods

### Cell lines

HCT116 cells with or without normal p53 expression (a gift from Dr. B. Vogelstein at the Johns Hopkins University, Baltimore, MD, USA) and human monocytic THP-1 cells were cultured in RPMI-1640 medium (Thermo Fisher Scientific, Waltham, MA, USA) supplemented with 10% fetal bovine serum (FBS) (Thermo Fisher Scientific), L-glutamine (2 mM) (Thermo Fisher Scientific), penicillin (100 units/ml) and streptomycin (100 μg/ml) (Thermo Fisher Scientific). The cells were maintained in a 37 °C incubator with 95% humidity and 5% CO_2_.

### Fucoidans

Low molecular weight Fucoidan (LMF) (also named as Oligo-Fucoidan) were prepared and characterized by Hi-Q Marine Biotech International Ltd. (New Taipei City, Taiwan) as previously described^28,54,59–61^. Oligo-Fucoidan (approximately molecular weights 0.5~0.8 kDa, 92.1%) was derived from glycolytic cleavage product of original Fucoidan from brown algae *Sargassum hemiphyllum*. The purified Oligo-Fucoidan (purity ≥ 98%) was mainly comprised of L-fucose (210.9 ± 3.3 μmol/g) and sulfate ester (38.9 ± 0.4% (w/w)). Oligo-Fucoidan was dissolved in double-distilled water at the indicated stock concentration (50 mg/ml), stirred at 65 °C, filter-sterilized through a 0.45-μm MF-Minipore membrane filter (EMD Millipore, Darmstadt, Germany) and stored at −20 °C. The high molecular weight Fucoidan (Sigma-F8190, purity ≥ 95%) (molecular weights 20~200 kDa) was purified from *Fucus vesiculosus* (Sigma-Aldrich, St. Louis, MO, USA).

### Antibodies (Abs)

Abs against phospho-ATM (Ser1981), ATM, phospho-Chk1 (Ser317), Chk1, phospho-Chk2 (Thr68), Chk2, phospho-p53 (Ser15), PTEN, phospho-CDK2 (Thr160), CDK2, Cyclin E, Cyclin B1, CDC2, phospho-CDC2 (Tyr15), cleaved PARP (Asp214), phospho-STAT3 (Ser727), STAT3, STAT5, caspase-3 and active caspase-3 (Asp175) were all obtained from Cell Signaling (Danvers, MA, USA). Abs recognizing p53 (EMD Millipore, Darmstadt, Germany), p21 (Santa Cruz, Dallas, Texas) and phospho-STAT5 (Ser726/731) (Sabbiotech, College Park, MD, USA) were obtained as indicated.

### Western blot analysis

The cells were incubated with CytoBuster^TM^ Protein Extraction Reagent (Novagen, Darmstadt, Germany), protease inhibitor cocktail (Sigma-Aldrich, MO, USA) and phosphatase inhibitor cocktail 2 (Sigma-Aldrich) for 30 min at 4 °C. The cell extracts were then purified by centrifugation at 12,000 rpm for 15 min at 4 °C, and protein concentrations were determined by Coomassie Plus^TM^ Protein Assay Reagent (Thermo Fisher Scientific). Protein samples (15 μg) were mixed with Tricine sample buffer (Protech Technology, Taipei, Taiwan), heated for 5 min at 95 °C, resolved on a 4–12% Bis/Tris NuPAGE gel (Invitrogen, Carlsbad, CA, USA) and transferred to a Nitrocellulose blotting membrane (Amersham Biosciences, Piscataway, NJ, USA) using a Trans-Blot SD Semi-Dry Electrophoretic Transfer Cell (Bio-Rad Laboratories, Hercules, CA, USA). The membranes was subsequently blocked with 5% Difco^TM^ Skim Milk (BD Biosciences, Franklin Lakes, NJ, USA) in PBS for 1 h, hybridized with the indicated Ab and then incubated with horseradish peroxidase (HRP)-conjugated sheep anti-mouse IgG or goat anti-rabbit IgG (Millipore, Billerica, MA, USA) for 1 h before being incubated with Immobilon^TM^ Western Chemiluminescent HRP Substrate (Millipore). The immuno-reactive signals were stripped by Restore^TM^ Western Blot Stripping Buffer (Thermo Fisher Scientific) before the protein of interest was re-probed.

### Mitochondrial superoxide levels

HCT116 cells were treated with Oligo-Fucoidan (400 μg/ml) and/or etoposide (40 μM) (Sigma-Aldrich) for 48 h and then incubated with 5 μM MitoSOX^TM^ reagent (Molecular Probes, Eugene, OR, USA) for 10 min at 37 °C. The emitted MitoSOX fluorescence was quantified by a FACSCalibur flow cytometer (Becton-Dickinson, Franklin Lakes, NJ, USA) on the FL2 emission channel at an excitation wavelength of 510 nm to measure the degree of MitoSOX Red oxidation.

### Cell cycle analysis

HCT116 cells (1 × 10^6^) were treated with Oligo-Fucoidan (400 μg/ml) and/or etoposide (40 μM) for 48 h. The cells were subsequently fixed with 70% ethanol at −20 °C for 1 h and incubated with 0.1% (v/v) Triton X-100 (Sigma-Aldrich), 5 μg/ml DNase-free RNase A (Sigma-Aldrich) and 10 μg/ml propidium iodide (PI) (Thermo Fisher Scientific) in PBS in the dark for 20 min at room temperature. PI fluorescence was analyzed by a FACSCalibur flow cytometer on the FL2 emission channel at an excitation wavelength of 488 nm.

### Enzyme-linked immunosorbent assay (ELISA) of IL-6 and CCL2

HCT116 cells were treated with etoposide and Oligo-Fucoidan or etoposide alone for 24 h. After the cells had incubated in serum-free media for 48 h, the concentrations of IL-6 and CCL2 in the conditional media were quantified by an ELISA kit (BOSTER Systems, Pleasanton, CA), according to the manufacturer’s instructions.

### Cell viability assay

Cell viability was determined by 3-(4,5-dimethylthiazol-2-yl)-2,5-diphenyltetrazolium bromide (MTT) assay (Sigma-Aldrich) after the cells had incubated with etoposide alone or etoposide and different concentrations of Oligo-Fucoidan for 48 h. The amount of tetrazolium dye converted to insoluble formazan by mitochondrial dehydrogenase was measured by a microplate reader (Tecan, Männedorf, Switzerland). The absorbance was measured at a wavelength of 570 nm.

### Quantitative real-time polymerase chain reaction (quantitative RT-PCR)

Cellular RNA was isolated by TRIzol^TM^III RNA solution (Thermo Fisher Scientific), after which DNase I-treated RNA samples were reverse-transcribed into cDNA using SuperScript^TM^III and oligo (dT) primers (Thermo Fisher Scientific). Quantitative RT-PCR was conducted using SYBR Green Master Mix and a LightCycler PCR detection system (ABI PRISM-7900). Each reaction contained a cDNA template (100 ng) and the indicated forward and reverse primers for IL-6 (forward: TCCAGTTGCCTTCTTGGGAC; reverse: GTACTCCAGAAGACCAGAGG), CCL2 (forward: AGGTGACTGGGGCATTGAT; reverse: GCCTCCAGCATGAAAGTCTC) and β-actin (forward: CACCAGGGCGTGATG GTGGG; reverse: GATGCCTCTCTTGCTCTGGGC), which were designed by the NCBI primer design program. PCR comprised the following steps: 95 °C for 15 min, followed by 40 cycles of 95 °C for 15 seconds and 60 °C for 1 min. The threshold cycles (∆C_t_) for the genes evaluated herein were normalized to those of the β-actin housekeeping gene (∆C_t_) via subtraction from a relative control value (∆∆C_t_). The fold-change in gene expression was calculated using the following formula: 2^−∆∆Ct^.

### Tumor growth in xenograft mice

Six-to-eight-week-old BALB/c nude mice (BALB/cAnN.Cg-Foxnlnu/CrlNarl) were obtained from the National Laboratory Animal Center of Taiwan. All experiments involving the mice were performed in accordance with the Animal Use Protocol approved by the National Health Research Institutes. The mice were injected subcutaneously with HCT116 cells (2 × 10^6^/100 μl PBS) before being randomized into 4 experimental groups (PBS, Oligo-Fucoidan, etoposide and the combined treatment of Oligo-Fucoidan and etoposide).

The maximum tolerated dose (MTD) of etoposide in mice is 40 mg/kg^62^. When the tumors reached approximately 100 mm^3^ in size, the mice were intraperitoneally (i.p.) injected with PBS or etoposide (8 mg/kg, 1/5 MTD) for 5 consecutive days and then fed Oligo-Fucoidan (300 mg/kg) or PBS orally thrice per week for 5 weeks so that the combined effects of the drugs could be assessed. Tumor volumes (Vs) were measured weekly and calculated using the following formula: Vs = 1/2 length × width^2^. Tumor-bearing mice were sacrificed and tumor burdens were analyzed.

### Immunohistochemistry (IHC)

The tumor tissues were fixed in 10% formalin (Sigma-Aldrich), embedded in paraffin and cut into 4-μm-thick sections before being de-paraffinized in xylene and hydrated in ethyl alcohol (100%, 95%, 85%, and 75%) for 7 min each. The tumor sections were then placed in citrate buffer (10 mM citric acid, 0.05% Tween 20, pH 6.0), heated for 20 min and rinsed twice with Tris-buffered saline-Tween (TBST, 0.05% Tween in TBS) for 3 min. The samples were then treated with 3% H_2_O_2_ in methanol for 15 min to block endogenous peroxidase activity before being rinsed with TBST for 3 min. We prevented non-specific binding by incubating the samples with 3% bovine serum albumin (Sigma-Aldrich) and IHC background blocker (Enzo Biochem, Inc., Farmingdale, NY, USA) in TBST for 30 min, reacting them with CD163 Ab (Abcam, ab189915, Cambridge, MA, USA) diluted in TBST (1:100) for 2 h and then incubating them with the secondary Ab (rabbit-on-rodent HRP-polymer) (Biocare Medical, Concord, CA, USA) for 1 h. The samples were then stained with a Liquid DAB + Substrate-Chromogen System (Dako Denmark A/S, Glostrup, Denmark) and counterstained with Gill No. 2 hematoxylin (Thermo Fisher Scientific). The slides were subsequently dehydrated, mounted with Histokitt mounting medium (Glaswarenfabrik Karl Hecht GmbH & CO., Germany) and observed under a Nikon Optiphot-2 Upright Microscope (Nikon Corporation, Tokyo, Japan) with a 40X objective lens. Images were photographed by a Nikon DXM1200 CCD digital camera and then analyzed under the same profile by Nikon ACT-1 imaging capture software.

### Statistics

Statistical analyses were performed using Student’s *t*-test, which was performed to compare the control and experimental groups. A *p* value < 0.05 was considered statistically significant.

## Electronic supplementary material

Supplementary figures

Supplementary Information
