# Supplementary material for: Oligo-Fucoidan prevents IL-6 and CCL2 production and cooperates with p53 to suppress ATM signaling and tumor progression
Source: Sci Rep. 2017 Sep 19;7:11864. doi: 10.1038/s41598-017-12111-1 (PMC5605496; doi:10.1038/s41598-017-12111-1)

Supplementary figures

Oligo-Fucoidan prevents IL-6 and CCL2 production and cooperates with p53 to suppress ATM signaling and tumor progression

Li-Mei Chen[1#], Po-Yen Liu[1#], Yen-An Chen[1], Hong-Yu Tseng[1], Pei-Chun Shen[1], Pai-An Hwang[2] and Hsin-Ling Hsu*

Supplementary Figure 1. The ATM signaling pathway is more suppressed in the $p53^{+/+}$ cells than the $p53^{-/-}$ cells after etoposide and Oligo-Fucoidan co-treatment. The effects of synchronized treatment with Oligo-Fucoidan (200 μg/ml) and etoposide (40 μM) for different intervals were assayed. The expression levels of different proteins were normalized to those β-actin, whose expression levels in MOCK-treated cells were defined as 1.

Supplementary Fig. 1

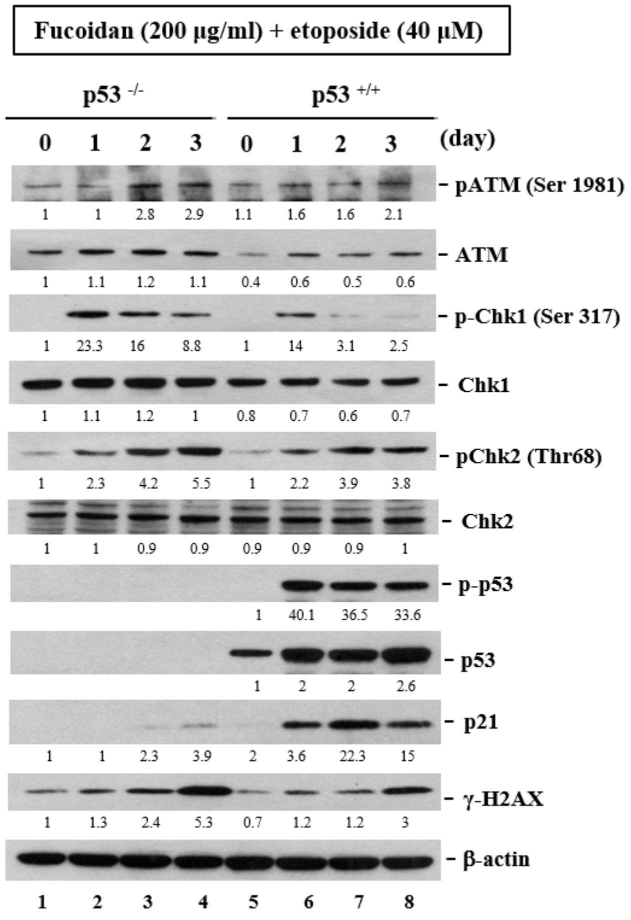

Oligo-Fucoidan prevents IL-6 and CCL2 production and cooperates with p53 to suppress ATM signaling and tumor progression

Li-Mei Chen[1#], Po-Yen Liu[1#], Yen-An Chen[1], Hong-Yu Tseng[1], Pei-Chun Shen[1], Pai-An Hwang[2] and Hsin-Ling Hsu*

Supplementary Figure 2. Oligo-Fucoidan (LMF) is more effective to suppress etoposide-induced DNA damage response than the HMF. (A) Different concentrations of the high molecular weight Fucoidan (HMF) from *Fucus vesiculosus* were examined in management of spontaneous DNA lesions in the HCT116 cells ($p53^{-/-}$ and $p53^{+/+}$). (B) The $p53^{-/-}$ cells were studied after treatment with etoposide (40 μM), Oligo-Fucoidan (LMF) (400 μg/ml) and the HMF (400 μg/ml) as well as co-treatment of LMF or HMF with etoposide for 48 h. The effects of LMF were compared with the HMF in response to etoposide treatment. (C) The $p53^{+/+}$ cells were studied after treatment with etoposide, LMF and the HMF as well as co-treatment of LMF or HMF with etoposide for 48 h. The expression levels of various proteins were normalized to those β-actin, whose expression levels in MOCK-treated cells were defined as 1.

Supplementary Fig. 2

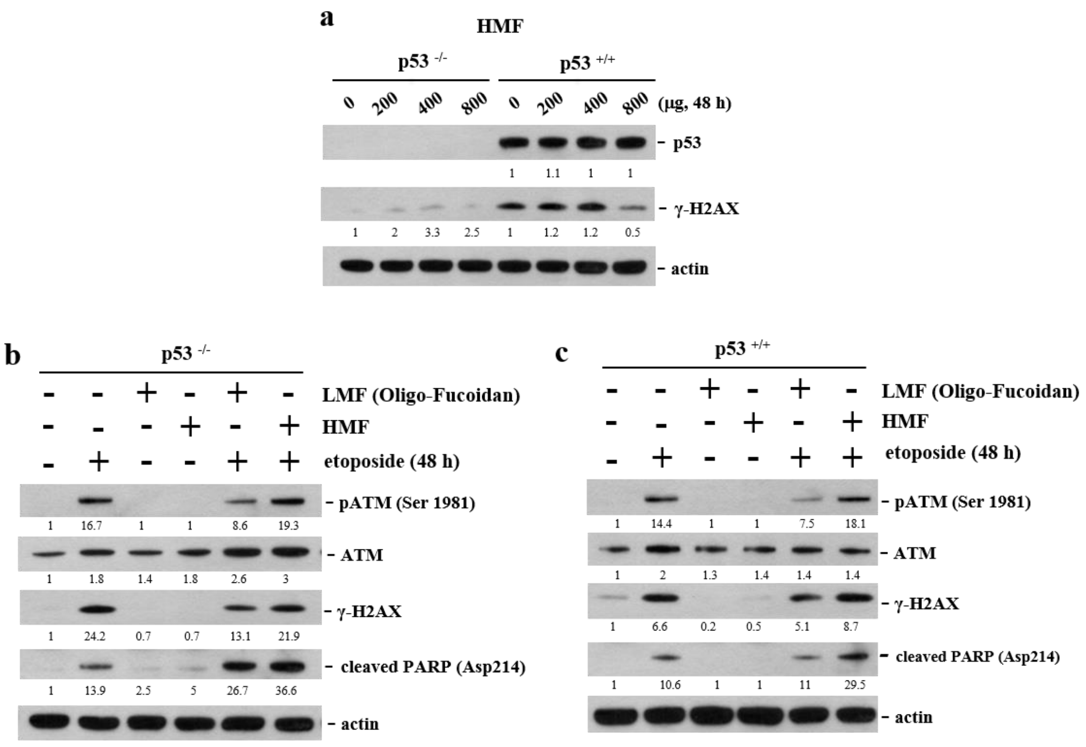

Supplement: Supplementary file 1 — Supplementary figures [file 41598_2017_12111_MOESM1_ESM.pdf]
