# Supplementary material for: Oligo-Fucoidan prevents IL-6 and CCL2 production and cooperates with p53 to suppress ATM signaling and tumor progression
Source: Sci Rep. 2017 Sep 19;7:11864. doi: 10.1038/s41598-017-12111-1 (PMC5605496; doi:10.1038/s41598-017-12111-1)
Supplement: Supplementary file 2 — Supplementary Information [file 41598_2017_12111_MOESM2_ESM.doc]

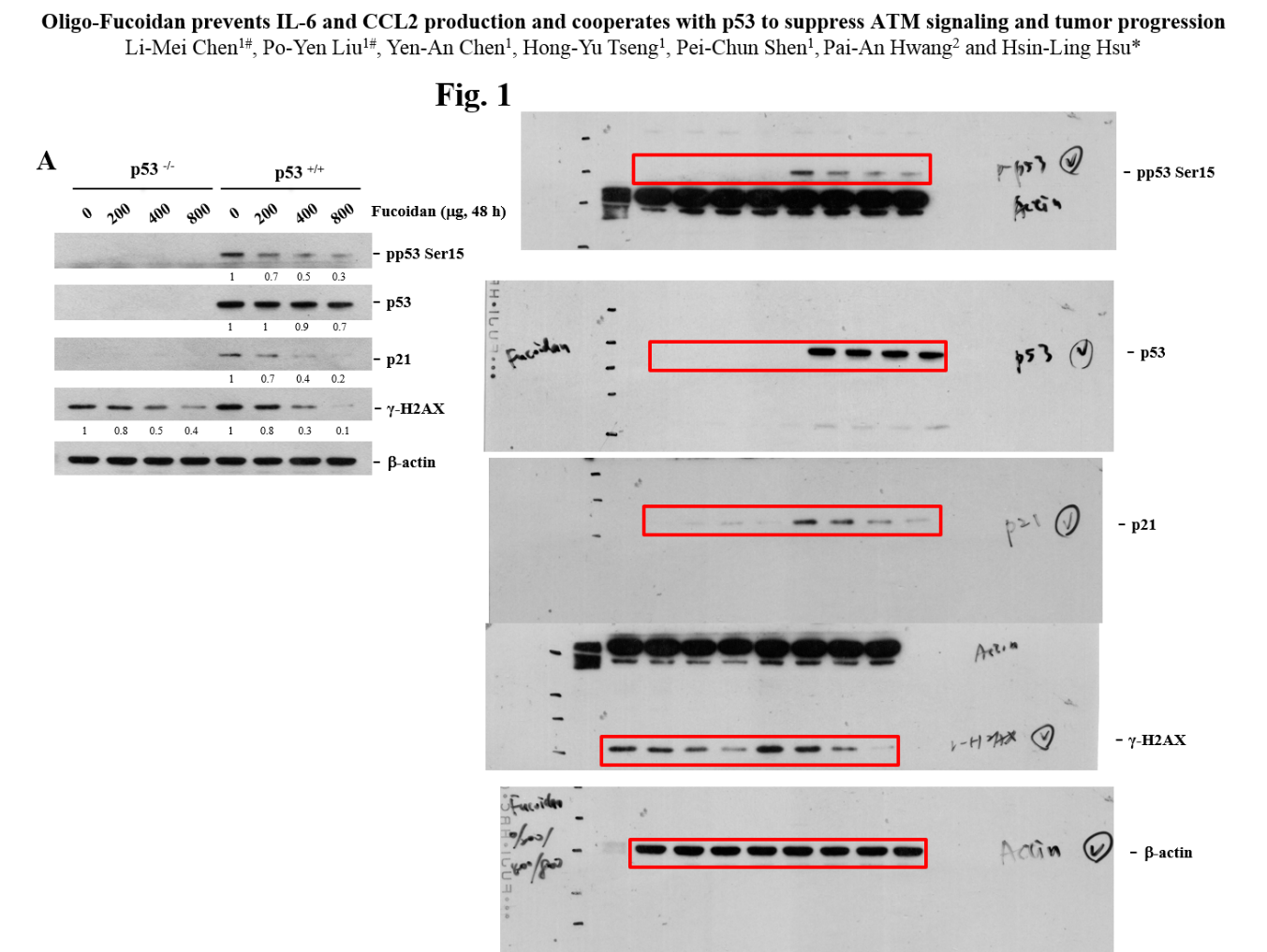


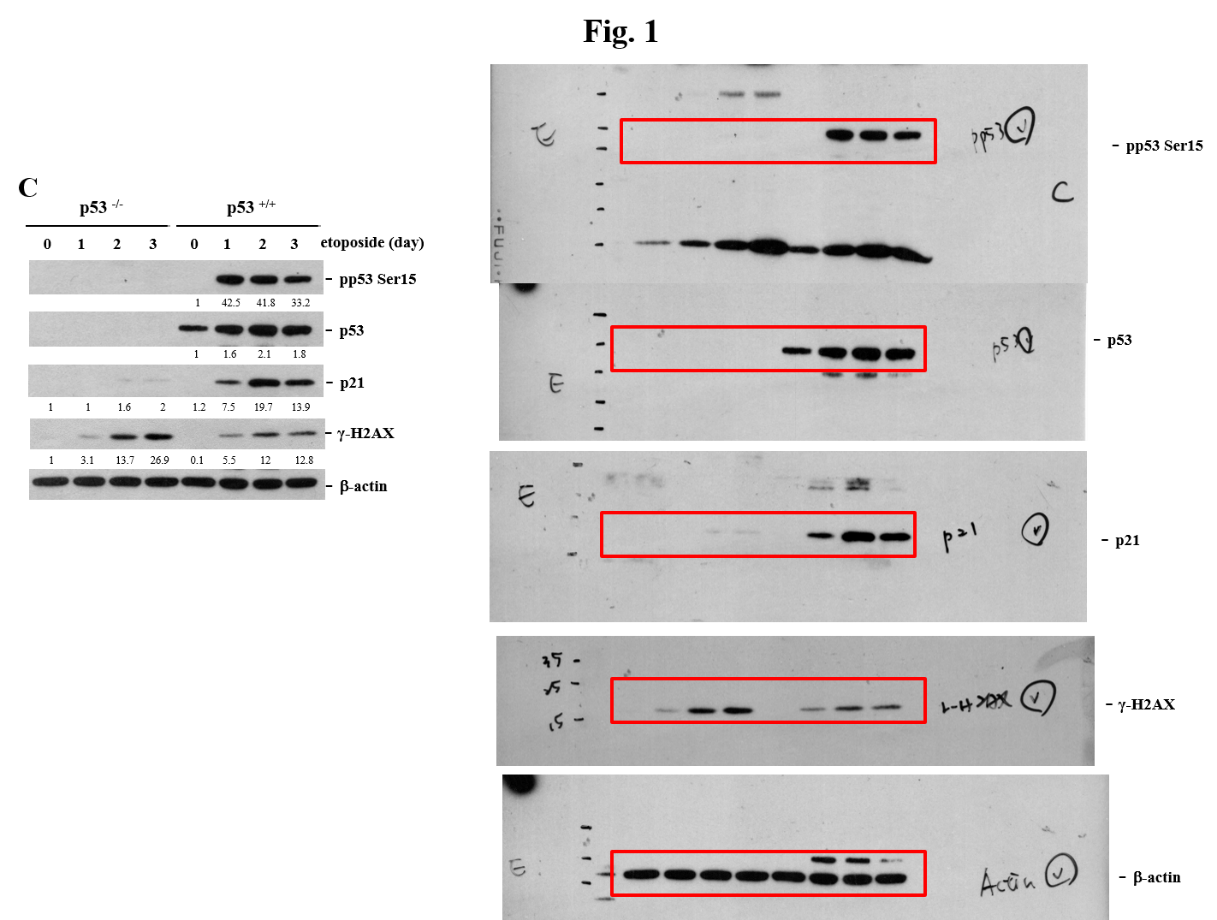


**Oligo-Fucoidan prevents IL-6 and CCL2 production and cooperates with p53 to suppress ATM signaling and tumor progression**

Li-Mei Chen^1#^, Po-Yen Liu^1#^, Yen-An Chen^1^, Hong-Yu Tseng^1^, Pei-Chun Shen^1^, Pai-An Hwang^2^

and Hsin-Ling Hsu*


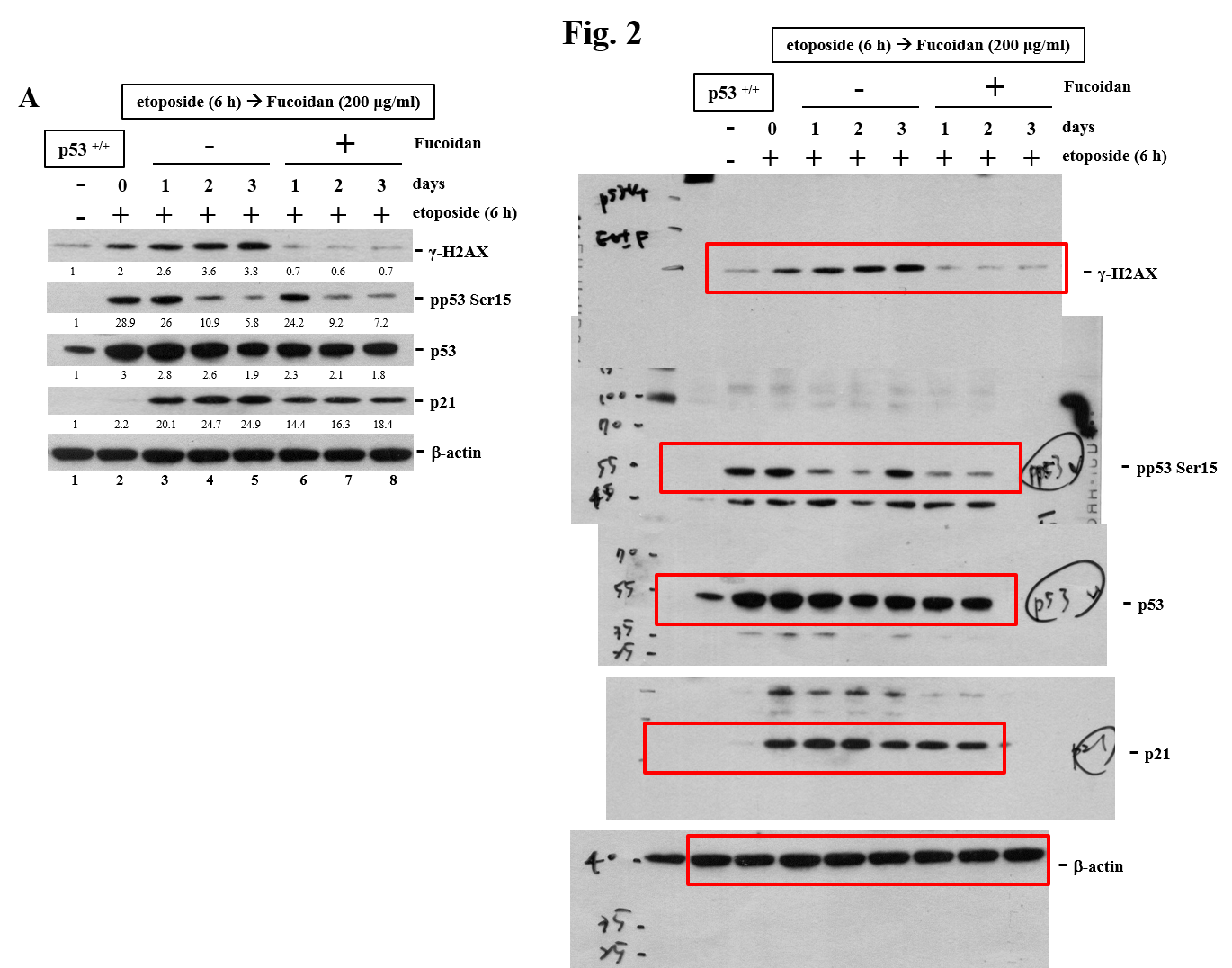


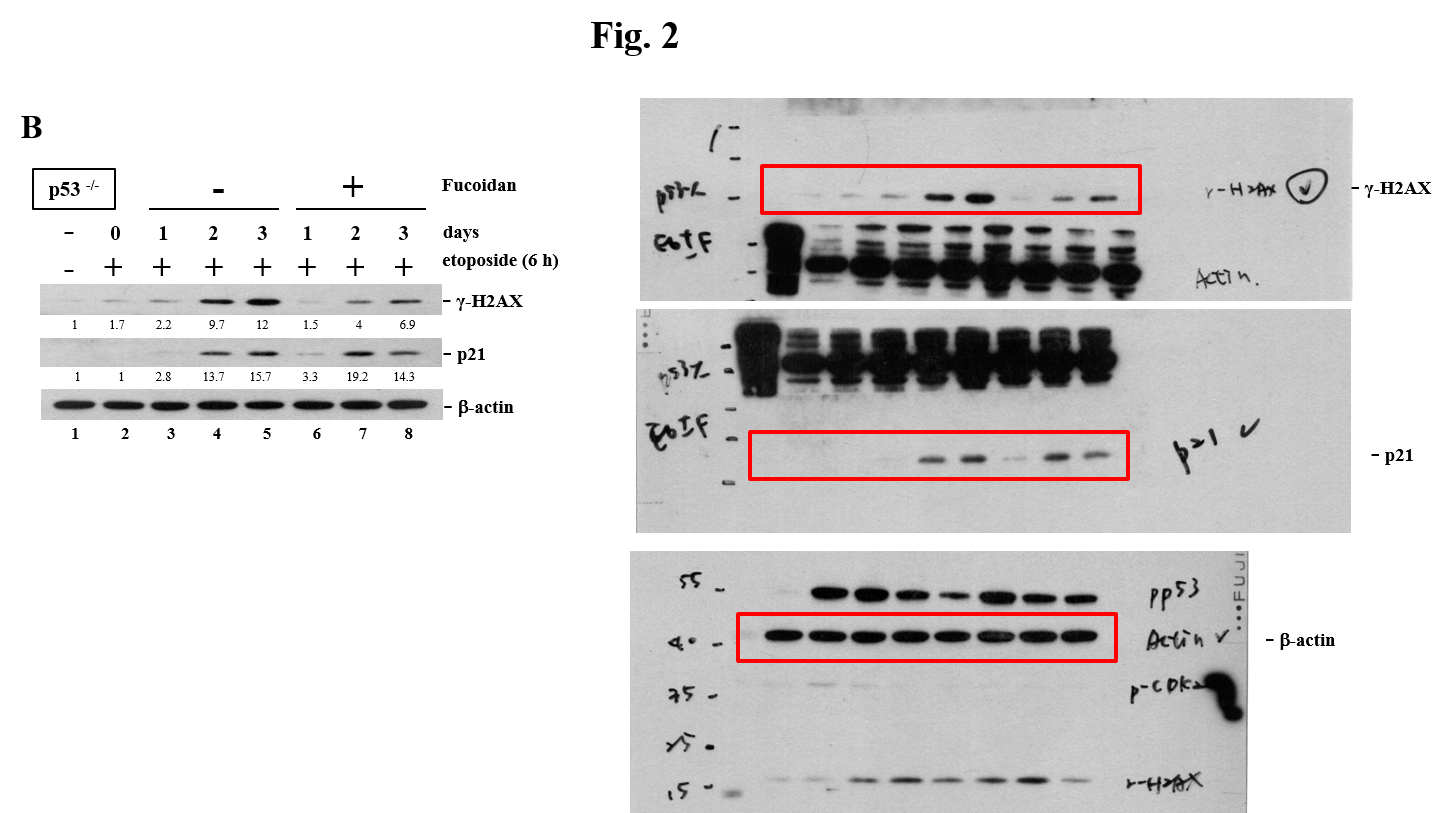


**Oligo-Fucoidan prevents IL-6 and CCL2 production and cooperates with p53 to suppress ATM signaling and tumor progression**

Li-Mei Chen^1#^, Po-Yen Liu^1#^, Yen-An Chen^1^, Hong-Yu Tseng^1^, Pei-Chun Shen^1^, Pai-An Hwang^2^

and Hsin-Ling Hsu*


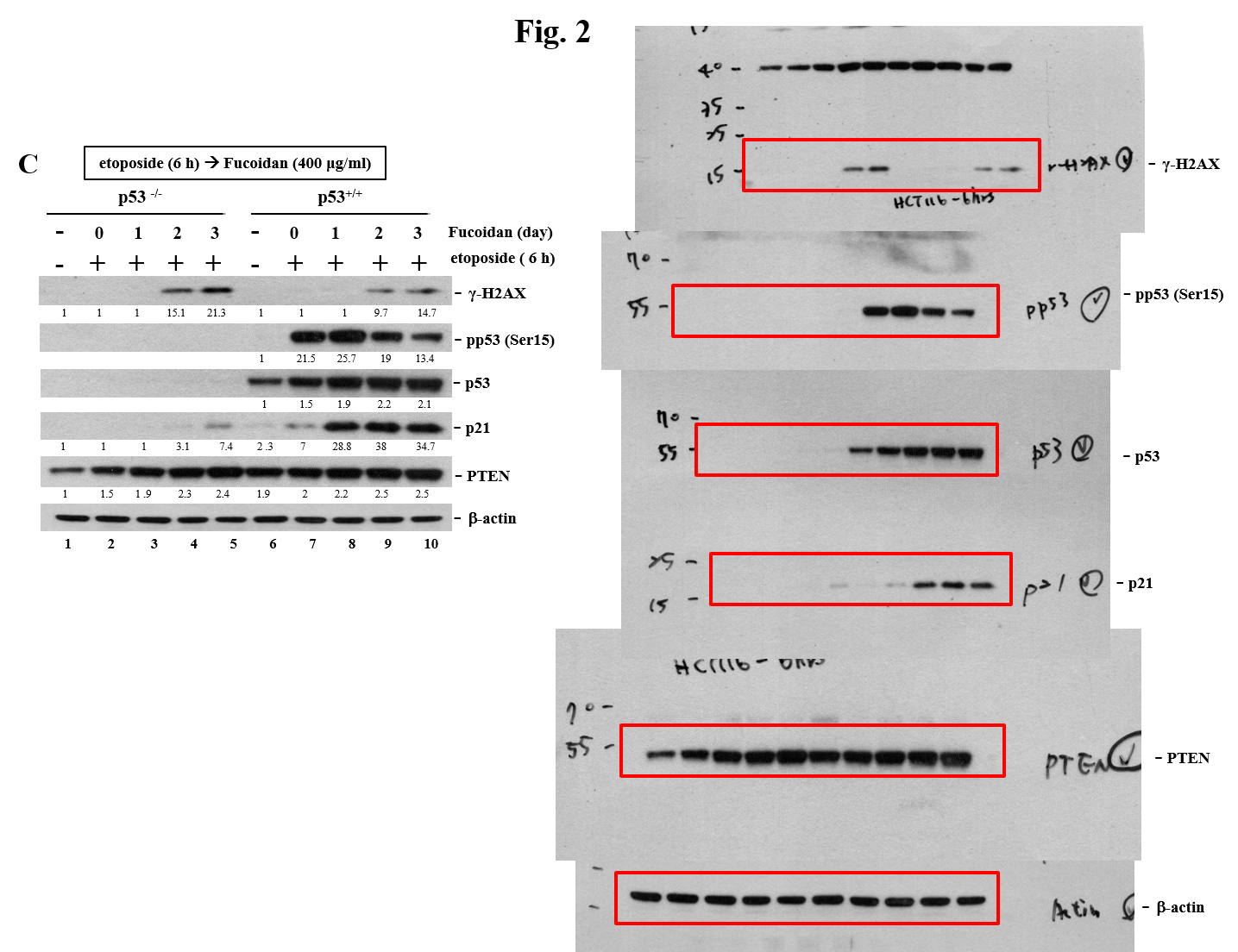


**Oligo-Fucoidan prevents IL-6 and CCL2 production and cooperates with p53 to suppress ATM signaling and tumor progression**

Li-Mei Chen^1#^, Po-Yen Liu^1#^, Yen-An Chen^1^, Hong-Yu Tseng^1^, Pei-Chun Shen^1^, Pai-An Hwang^2^

and Hsin-Ling Hsu*


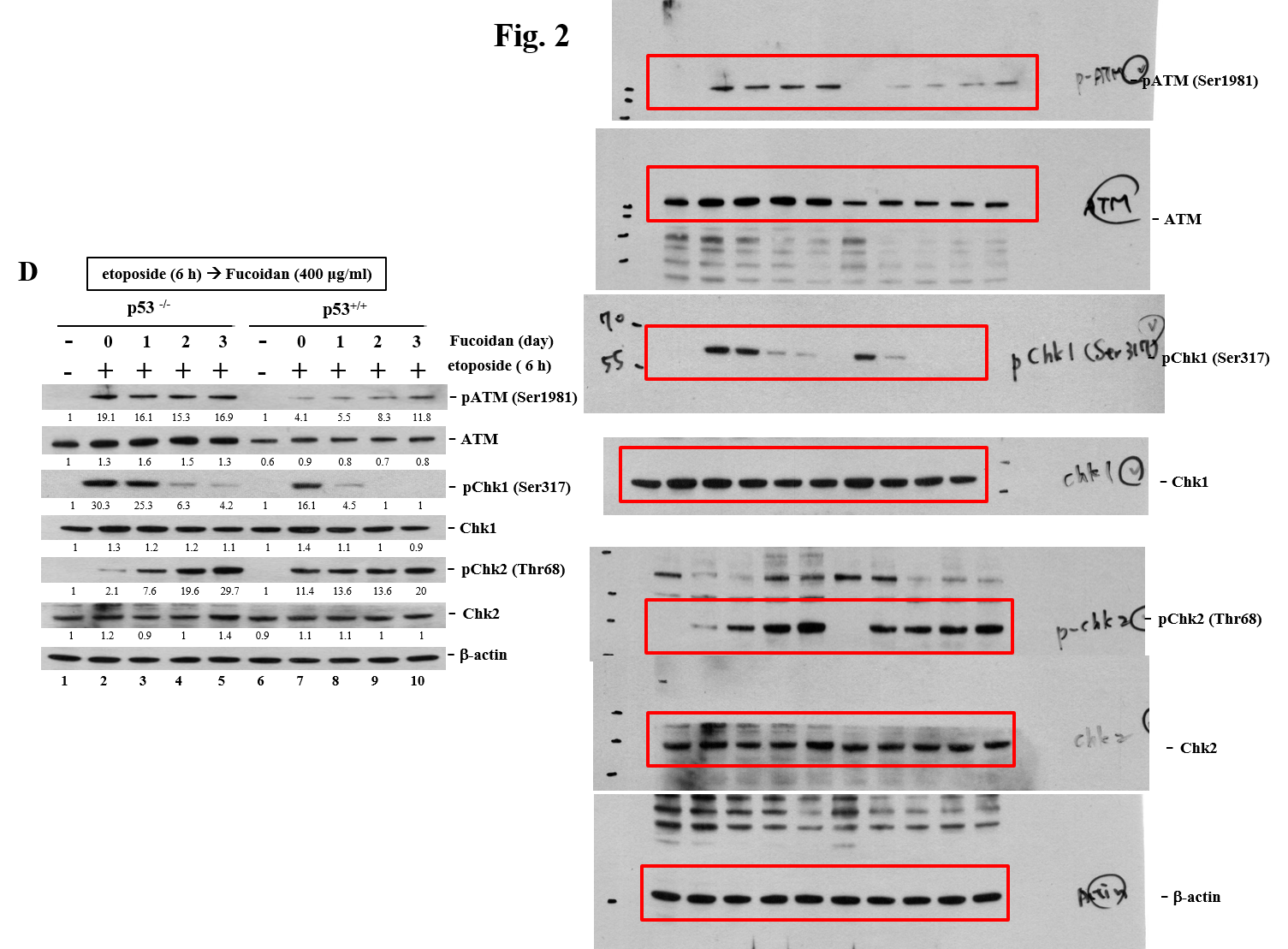


**Oligo-Fucoidan prevents IL-6 and CCL2 production and cooperates with p53 to suppress ATM signaling and tumor progression**

Li-Mei Chen^1#^, Po-Yen Liu^1#^, Yen-An Chen^1^, Hong-Yu Tseng^1^, Pei-Chun Shen^1^, Pai-An Hwang^2^

and Hsin-Ling Hsu*


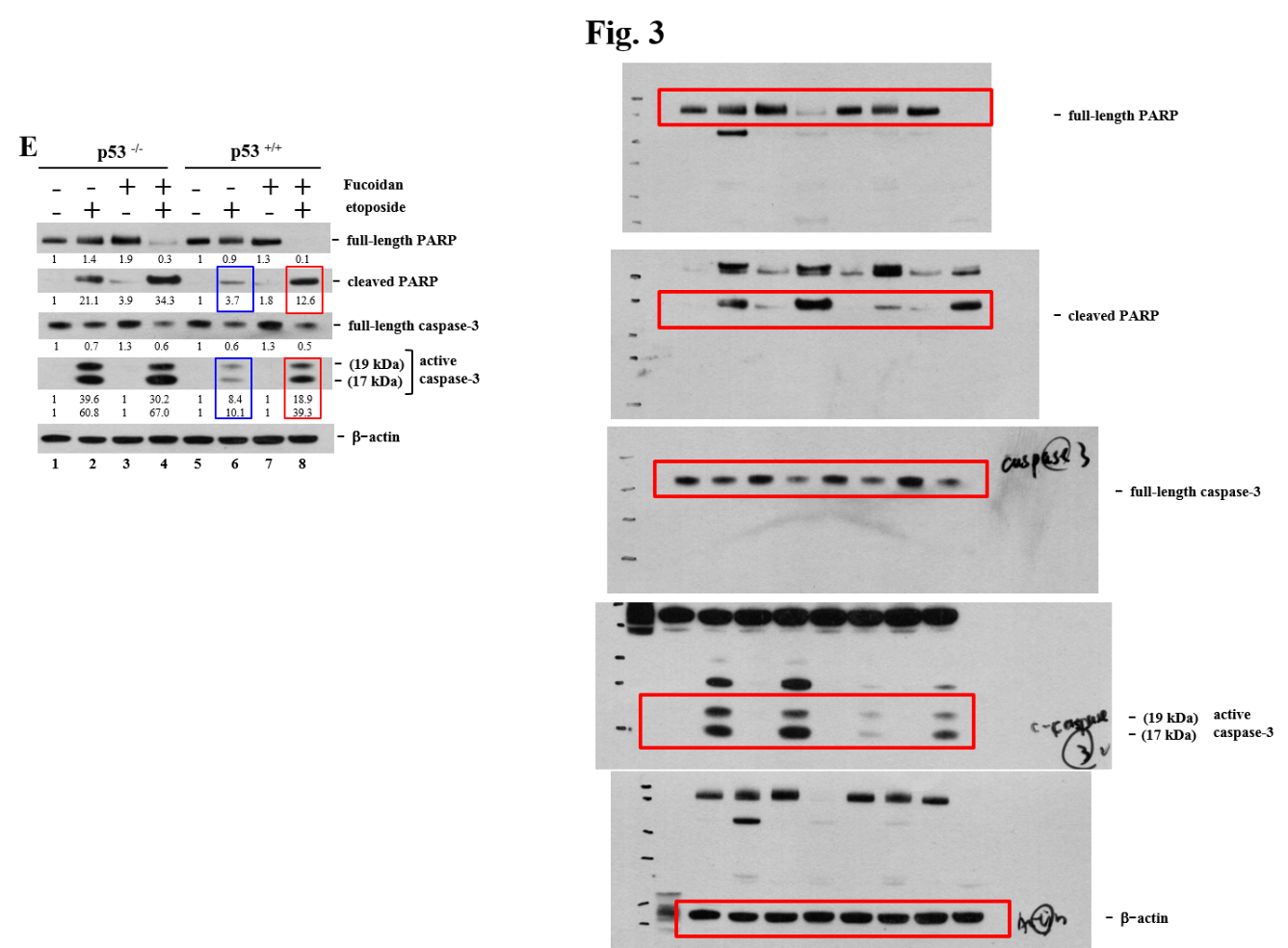


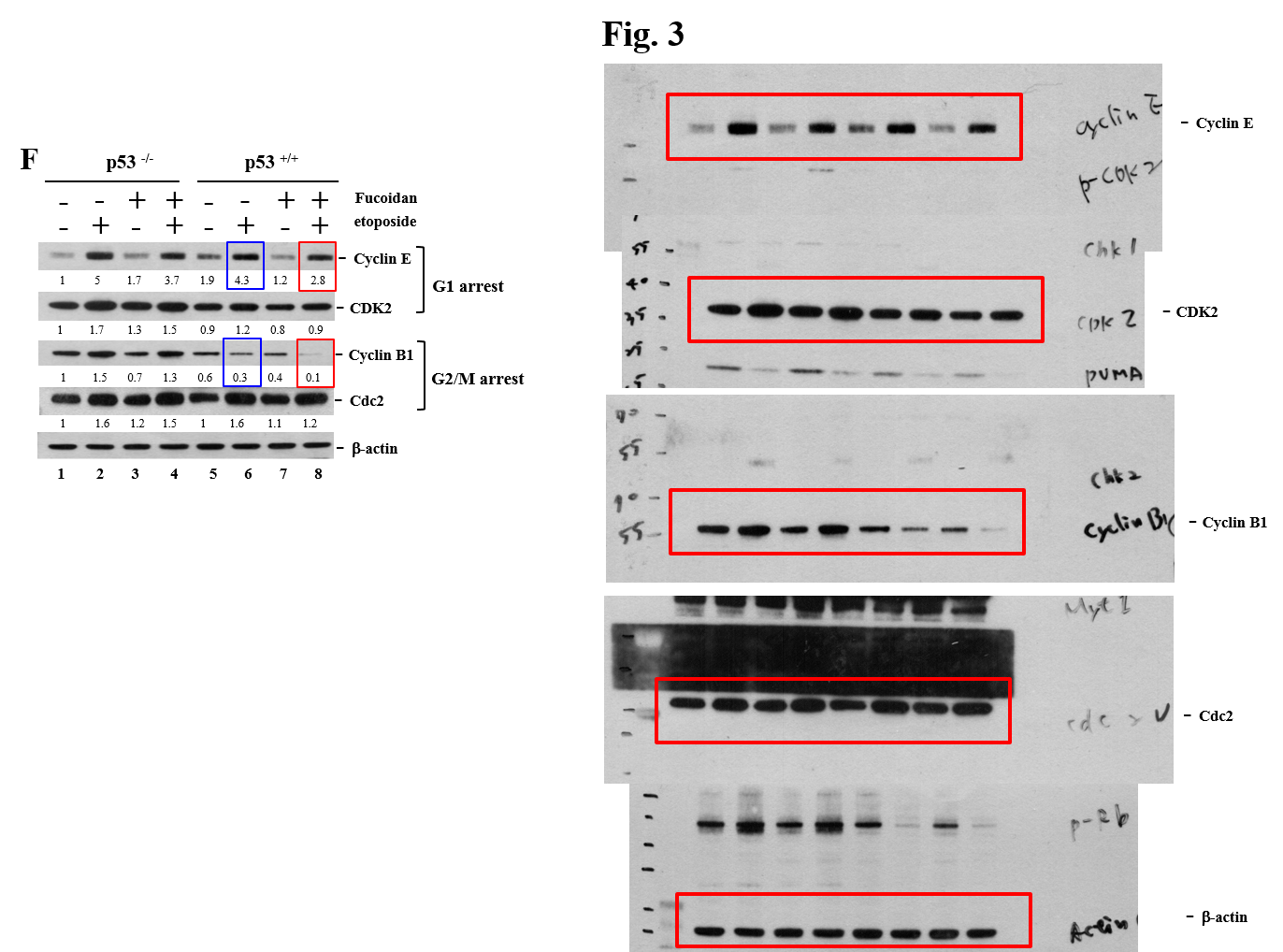


**Oligo-Fucoidan prevents IL-6 and CCL2 production and cooperates with p53 to suppress ATM signaling and tumor progression**

Li-Mei Chen^1#^, Po-Yen Liu^1#^, Yen-An Chen^1^, Hong-Yu Tseng^1^, Pei-Chun Shen^1^, Pai-An Hwang^2^

and Hsin-Ling Hsu*


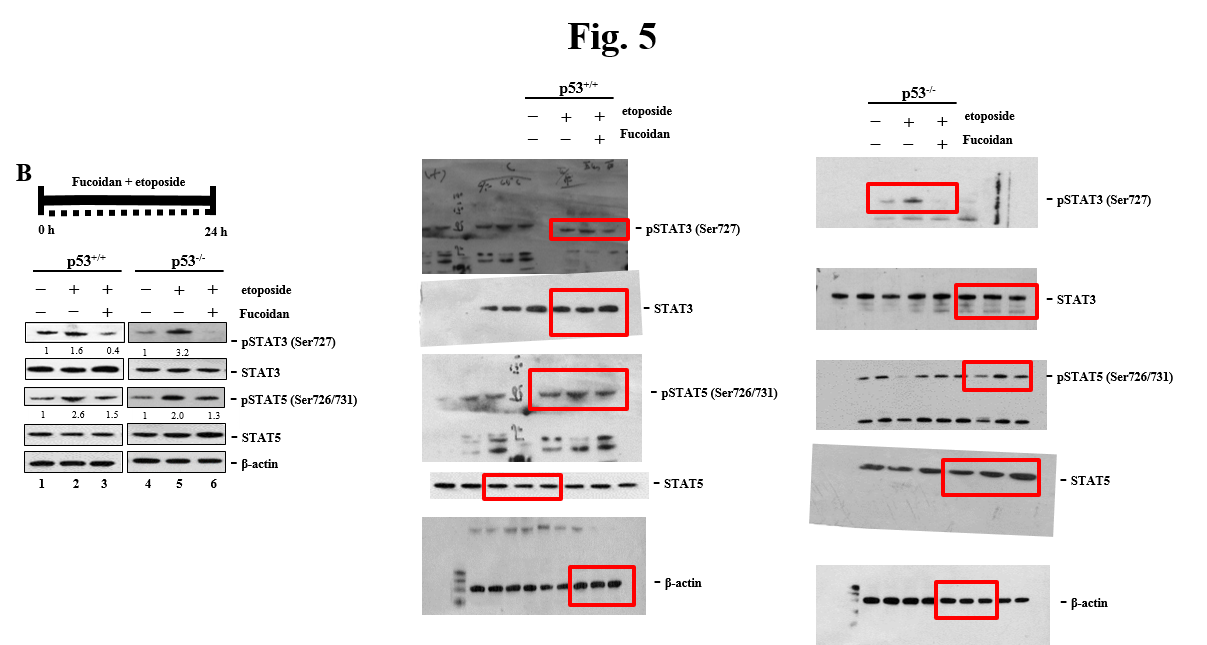


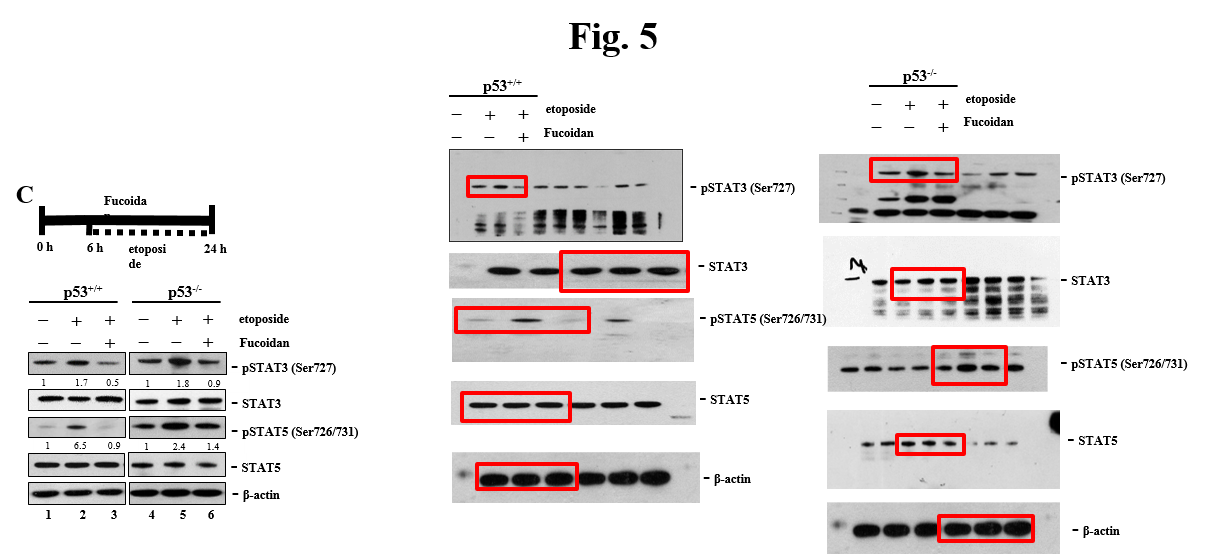


**Oligo-Fucoidan prevents IL-6 and CCL2 production and cooperates with p53 to suppress ATM signaling and tumor progression**

Li-Mei Chen^1#^, Po-Yen Liu^1#^, Yen-An Chen^1^, Hong-Yu Tseng^1^, Pei-Chun Shen^1^, Pai-An Hwang^2^

and Hsin-Ling Hsu*


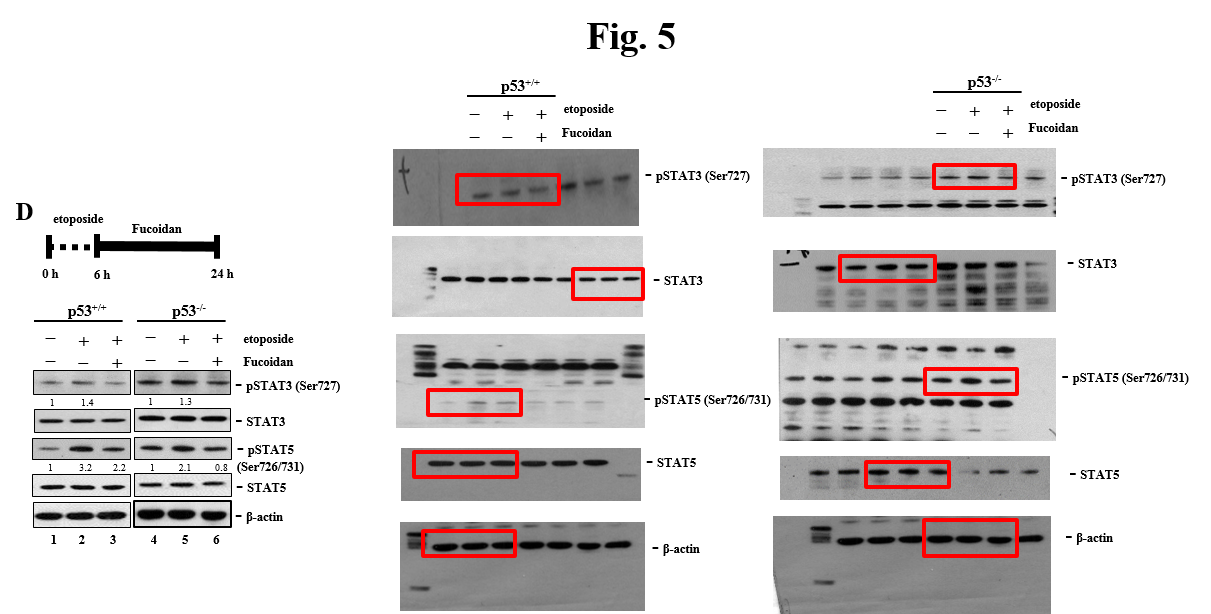


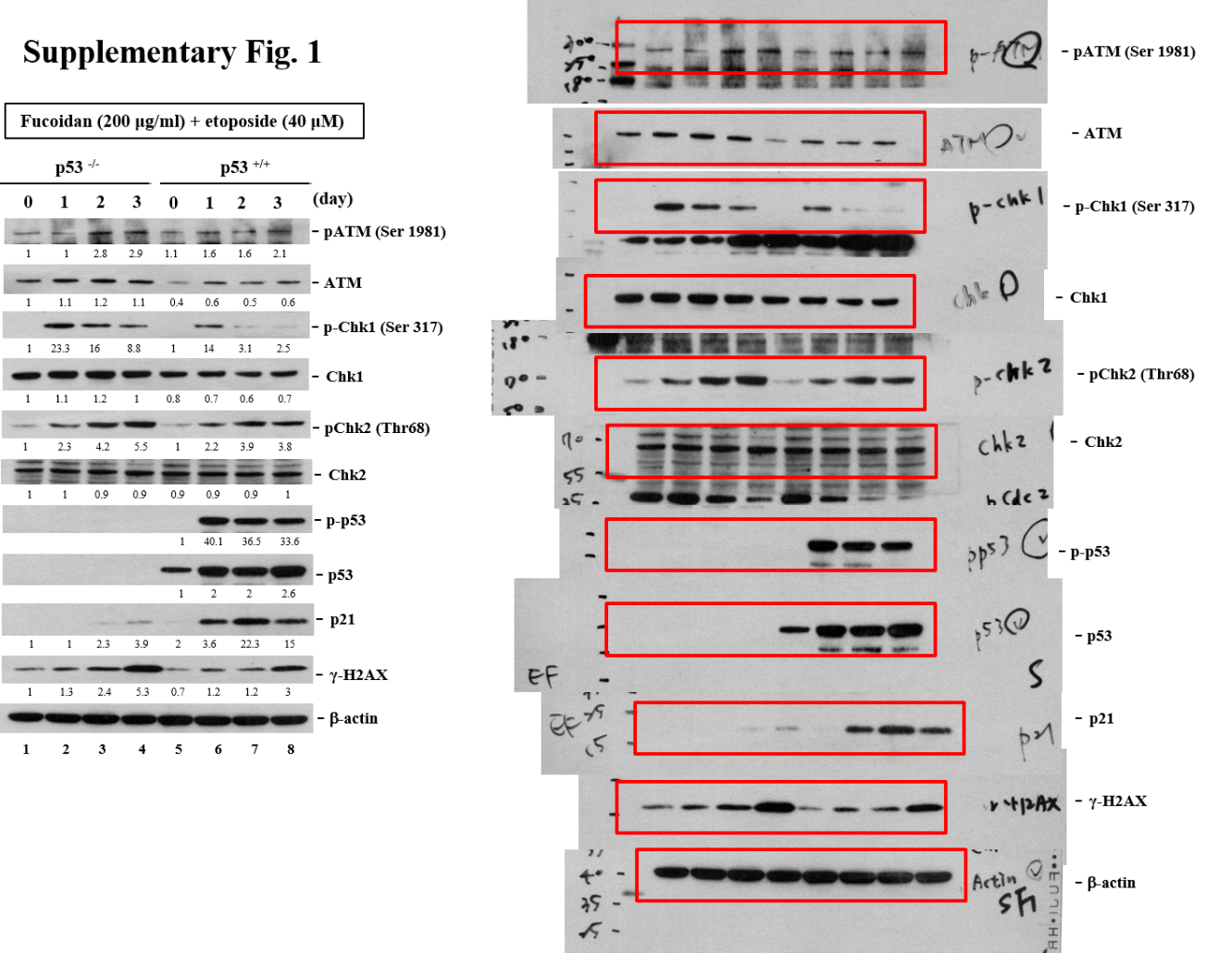


**Oligo-Fucoidan prevents IL-6 and CCL2 production and cooperates with p53 to suppress ATM signaling and tumor progression**

Li-Mei Chen^1#^, Po-Yen Liu^1#^, Yen-An Chen^1^, Hong-Yu Tseng^1^, Pei-Chun Shen^1^, Pai-An Hwang^2^

and Hsin-Ling Hsu*


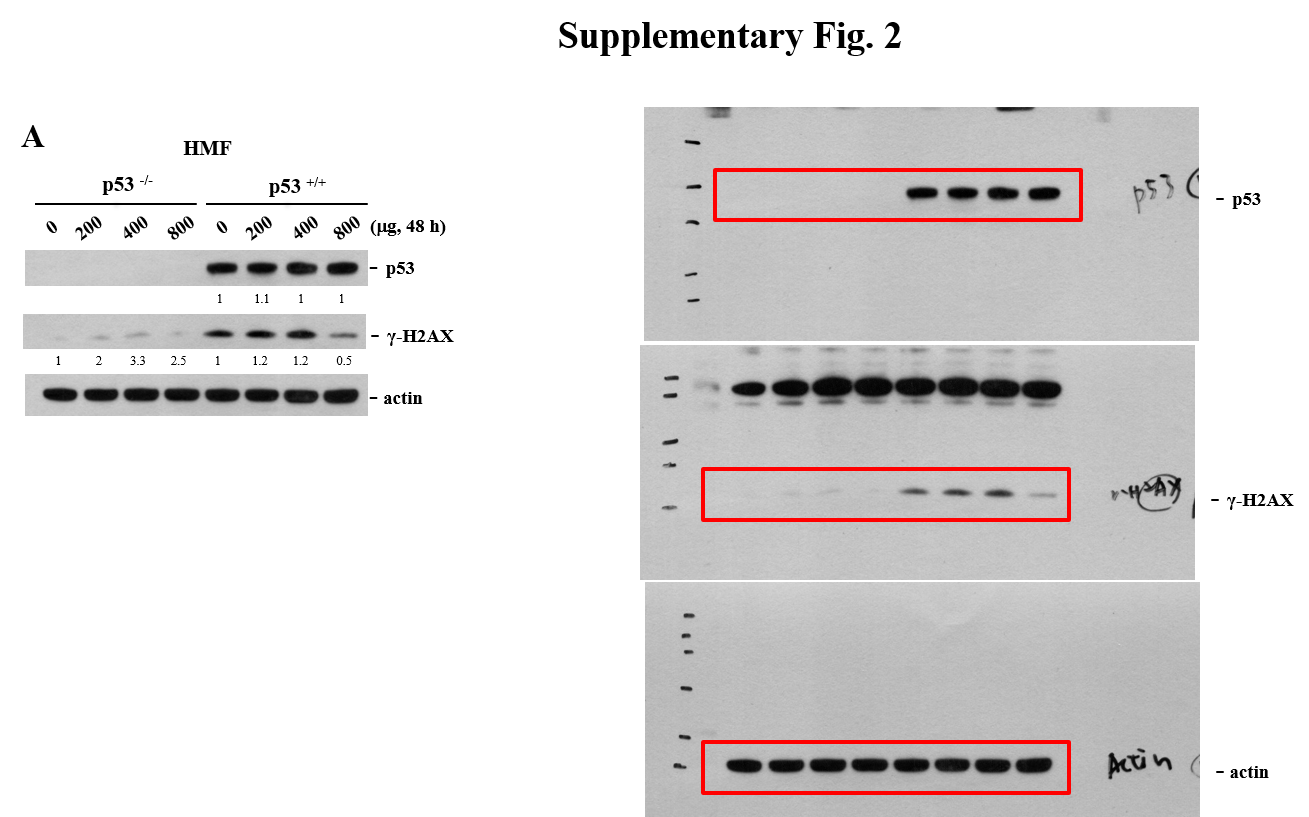


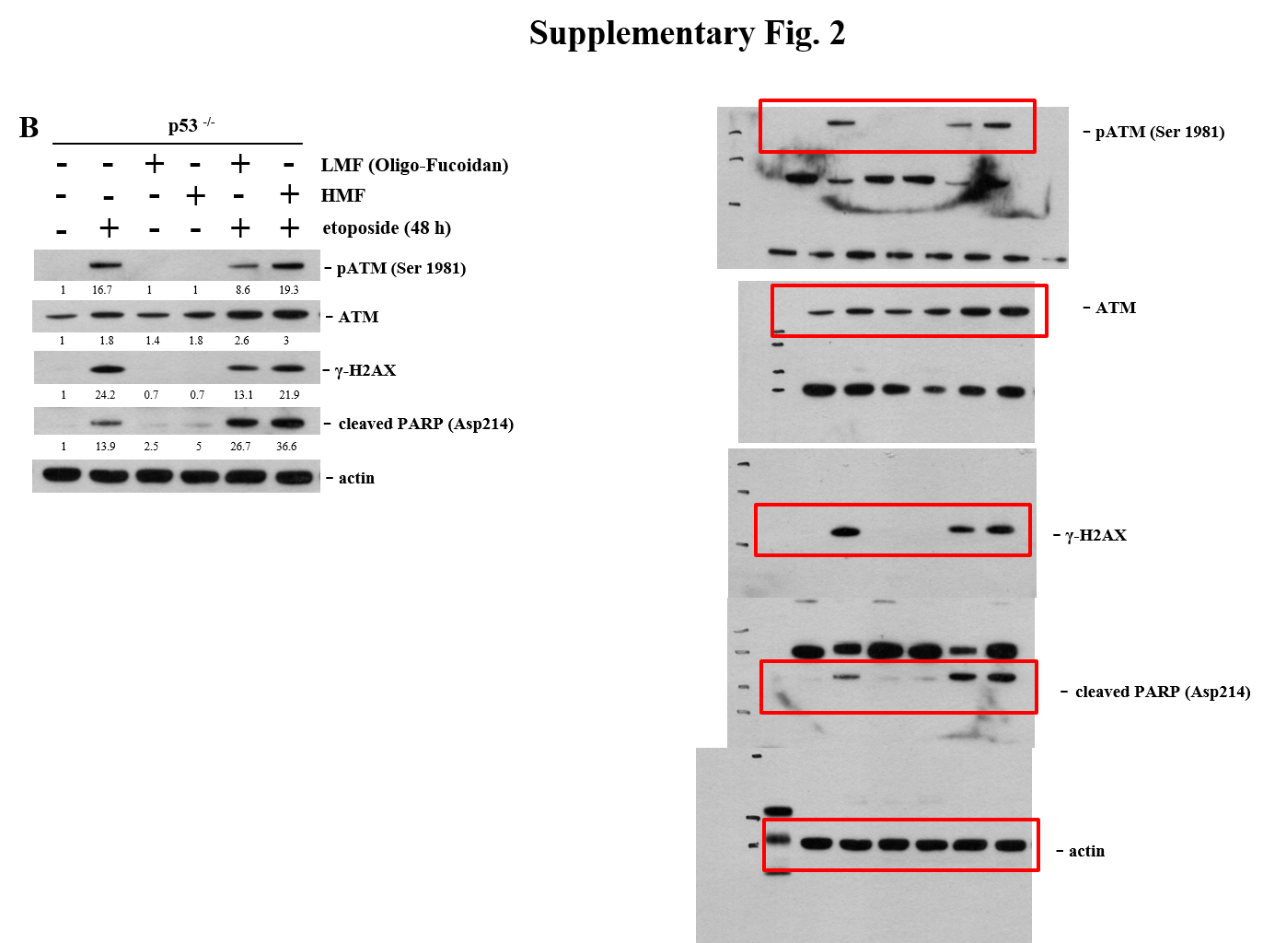


**Oligo-Fucoidan prevents IL-6 and CCL2 production and cooperates with p53 to suppress ATM signaling and tumor progression**

Li-Mei Chen^1#^, Po-Yen Liu^1#^, Yen-An Chen^1^, Hong-Yu Tseng^1^, Pei-Chun Shen^1^, Pai-An Hwang^2^

and Hsin-Ling Hsu*


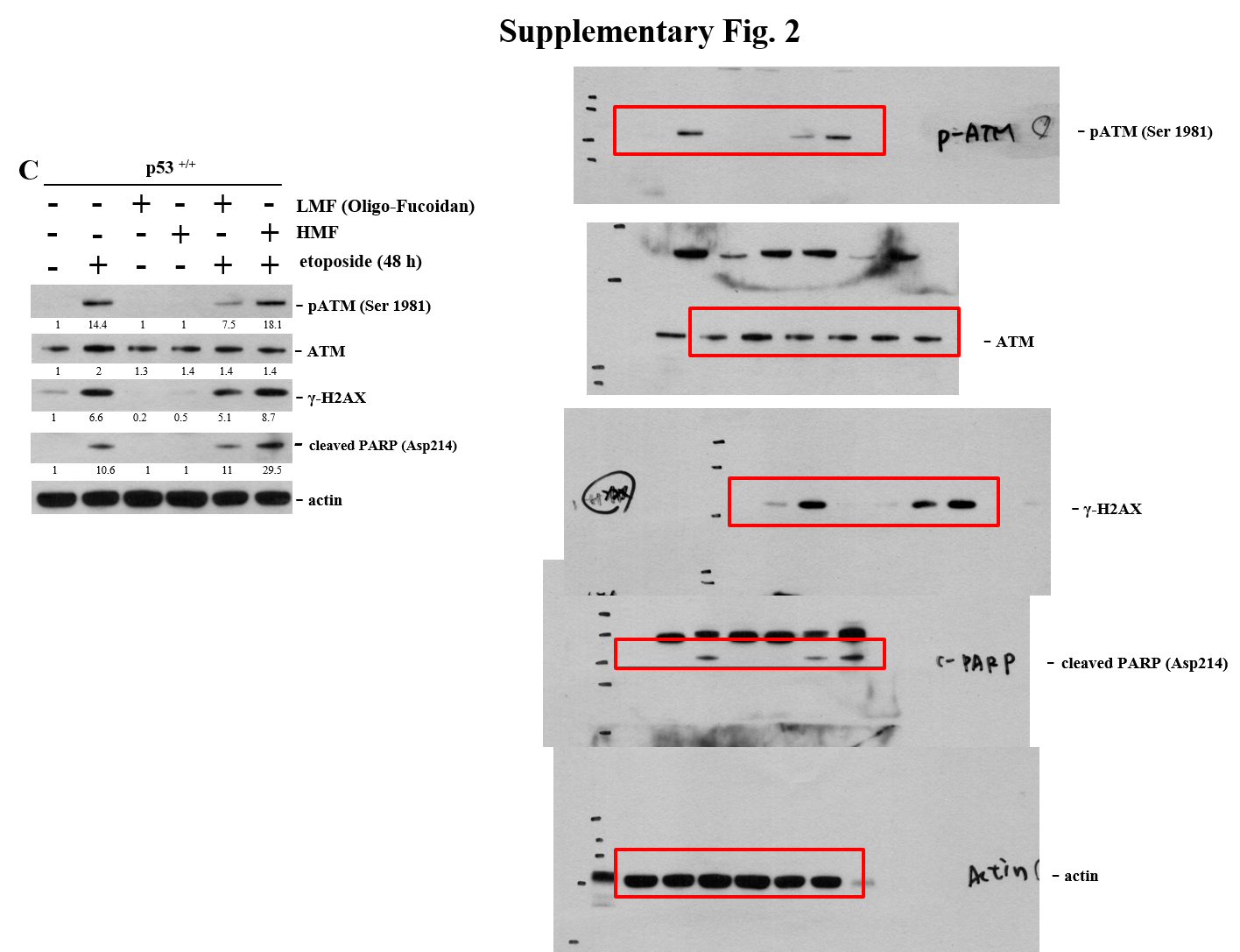


**Oligo-Fucoidan prevents IL-6 and CCL2 production and cooperates with p53 to suppress ATM signaling and tumor progression**

Li-Mei Chen^1#^, Po-Yen Liu^1#^, Yen-An Chen^1^, Hong-Yu Tseng^1^, Pei-Chun Shen^1^, Pai-An Hwang^2^

and Hsin-Ling Hsu*
